# Metabolic modulations of *Pseudomonas graminis* in response to H_2_O_2_ in cloud water

**DOI:** 10.1038/s41598-019-49319-2

**Published:** 2019-09-05

**Authors:** Nolwenn Wirgot, Marie Lagrée, Mounir Traïkia, Ludovic Besaury, Pierre Amato, Isabelle Canet, Martine Sancelme, Cyril Jousse, Binta Diémé, Bernard Lyan, Anne-Marie Delort

**Affiliations:** 1 Université Clermont Auvergne, CNRS, Sigma-Clermont, Institut de Chimie de Clermont-Ferrand, F-63000 Clermont-Ferrand, France; 2 Plateforme d’Exploration du Métabolisme, Université Clermont Auvergne & I.N.R.A site de Theix, Clermont-Ferrand, France

**Keywords:** Metabolomics, Air microbiology, Atmospheric chemistry

## Abstract

In cloud water, microorganisms are exposed to very strong stresses especially related to the presence of reactive oxygen species including H_2_O_2_ and radicals, which are the driving force of cloud chemistry. In order to understand how the bacterium *Pseudomonas graminis* isolated from cloud water respond to this oxidative stress, it was incubated in microcosms containing a synthetic solution of cloud water in the presence or in the absence of H_2_O_2_. *P*. *graminis* metabolome was examined by LC-MS and NMR after 50 min and after 24 hours of incubation. After 50 min, the cells were metabolizing H_2_O_2_ while this compound was still present in the medium, and it was completely biodegraded after 24 hours. Cells exposed to H_2_O_2_ had a distinct metabolome as compared to unexposed cells, revealing modulations of certain metabolic pathways in response to oxidative stress. These data indicated that the regulations observed mainly involved carbohydrate, glutathione, energy, lipid, peptides and amino-acids metabolisms. When cells had detoxified H_2_O_2_ from the medium, their metabolome was not distinguishable anymore from unexposed cells, highlighting the capacity of resilience of this bacterium. This work illustrates the interactions existing between the cloud microbial metabolome and cloud chemistry.

## Introduction

Clouds are oxidative environments with a redox potential of up to more than 200 mV^1^. The oxidant capacity of the atmosphere is often evaluated based on the concentration of H_2_O_2_ in the gas and aqueous phases of clouds: this is highly variable depending on the season, day-night cycles, and the level of air pollution^2^. According to Deguillaume *et al*.^1^, the concentration of H_2_O_2_ can vary from 0 to 58 µM in cloud waters collected at the puy de Dôme Mountain station. H_2_O_2_ is the main source of hydroxyl radicals in clouds^3^; this oxidizes the organic matter (OM) into molecules such as organic acids or aldehydes and, ultimately, to CO_2_. Short chain organic acids and aldehydes account for most of the identified carbon compounds in cloud waters (about 10% of the OM). The most abundant are acetate, formate, succinate, malonate, oxalate and formaldehyde^1,4^. More recently, amino acids have been quantified to represent about 9% of the OM^5^ in clouds. Consistently, carboxylic acids, aldehydes and amino acids are also present at high concentrations in fog and aerosols^4,6,7^. The precise composition of the rest of the OM in the atmosphere is still not well defined. Recent investigation using FT-ICR-MS approaches provided a global description based on exact mass formulaes and H/C/O/N molecular ratios^8,9^.

Although cloud water is probably a stressful environment for living organisms, in particular due to the presence of reactive oxygen species (ROS) such as H_2_O_2_, it was shown that cloud microorganisms largely survive exposure to H_2_O_2_ at its natural atmospheric concentration^10,11^ and that some remain alive and metabolically active in clouds^12–15^. ATP (Adenosine TriPhosphate) is currently used as a proxy for metabolic activity and it was quantified in cloud waters^16–19^. Different studies have shown that airborne microorganisms are able to biotransform mono and dicarboxylic acids (oxalic, malonic, succinic, glutaric, adipic, pimelic, pinic acids, acetic, formic acids) and also formaldehyde and methanol^16,19–25^. The measured biotransformation rates were shown to be competitive with rates involving radical reactions. It was thus proposed that microbial activity could be an alternative route to radical chemistry^3,4,15,26,27^. In addition, cloud microorganisms impact H_2_O_2_ concentration by detoxifying it into H_2_O and O_2_ through catalases acivity, and consequently they can indirectly limit the efficiency of radical chemistry^11,19^.

The ability to manage ROS is a permanent challenge for aerobic organisms that use molecular oxygen as the terminal electron acceptor. Reactive by-products of oxygen, such as superoxide anion radical, hydroxyl radicals and hydrogen peroxide, are generated in aerobically grown cells. Oxidative stress is defined as an imbalance in favor of the prooxidants and disfavoring the antioxidants^28^; resulting in cellular damages damage (DNA, lipids, proteins and carbohydrates). To avoid such damages, microorganisms deploy various strategies involving specific enzyme like catalases, superoxide dismutases and the production of oxidant scavengers (glutathione, pigments, etc.). In addition, bacteria exposed to ROS are suspected to reorganize their entire functioning under oxidative stress. Modulation of the global cell metabolism can be observed using metabolomics. This powerful tool has been used for investigating oxidative stress response in plants^29^ and bacteria^30,31^.

In a recent study, we observed that H_2_O_2_ modulated the global metabolic functioning of microorganisms in cloud water, as shown by strong correlations between H_2_O_2_ and cellular ATP concentrations^11^. The objective of this work was to go further in elucidating precisely the metabolic modifications involved, with the aim tounderstand how cloud microorganisms may interact with cloud chemistry under very oxidative conditions. Since microorganisms use or produce organic compounds modulations of their metabolism can potentially influence the reactivity of organic compounds in clouds.

We used a metabolomics approach to investigate the response to H_2_O_2_ exposure of *Pseudomonas graminis* strain PDD-13b-3, a bacterium that we previously isolated from cloud water and whose genome is sequenced^32^. *Pseudomonas* species are among the most frequent bacteria observed in clouds, and they account for some of the most active cells in these environmnents^13,18^.

The combination of NMR and LC-MS analyses allowed identifying and discriminating the metabolites and the metabolic pathways impacted by this stress. Because our main objective was to highlight the modifications of the metabolism when the impact of H_2_O_2_ was maximum, we monitored the ATP concentration over time, and we sampled cells for metabolomics study at the time corresponding to the lowest ATP level (t = 50 min), when H_2_O_2_ was not totally degraded yet. We also investigated the metabolic status of the cells after 24 hours, when cells had recovered initial ATP concentration and no H_2_O_2_ was left. The interactions between the bacterial metabolome and cloud chemistry implied under these conditions are further discussed.

## Results

### Monitoring of hydrogen peroxide and ATP concentrations

**Figure 1:**
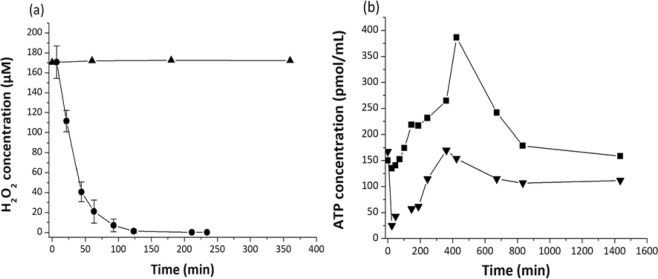
(**a**) Temporal evolution of H_2_O_2_ concentration in the presence (circles) or absence of *P*. *graminis* 13b-3 (triangles). (**b**) Evolution of ATP concentration in *P*. *graminis* 13b-3 incubated with H_2_O_2_ (triangles) or without H_2_O_2_ (squares). Error bars are standard errors of the means of three replicates. Where error bars are not visible, they are smaller than the symbol.

H_2_O_2_ degradation by *Pseudomonas graminis* PDD-13b-3 was monitored during the incubation by measuring its concentration until it was exhausted (Fig. 1a); H_2_O_2_ was completely biodegraded after two hours, and the corresponding degradation rate was on the same order of magnitude as compared to Wirgot *et al*.^11^ (in the range of 10^−17^ mol.min^−1^.cell^−1^).

The ATP concentration was closely monitored during the first 13 hours of incubation, and after 24 hours (Fig. 1b). The addition of H_2_O_2_ (time “0”) caused a rapid decrease of ATP concentration (170 pM to 25 pM in approximately 30 min), and this lasted until H_2_O_2_ was totally degraded (represented on the graph with a vertical bar around 120 min). After 400 min, *P*. *graminis* 13b-3 recovered an ATP concentration that was close to the initial value (approximately 150 pM). After 24 hours, the ATP concentration reached a concentration of 125 pM in all samples, indistinctly between cells exposed or unexposed to hydrogen peroxide.

To assess these two different response phases, the cell extract contents (i.e. the metabolomes) after 50 min and 24 h of incubation in the presence and in the absence of added H_2_O_2_ were compared.

### Impact of H_2_O_2_ exposure on *P*. *graminis* metabolome

#### Effect of exposure time on metabolic profile

**Figure 2:**
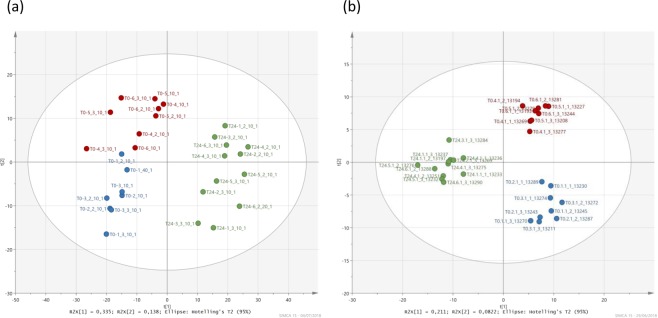
PLS-DA analyses (UV scaling). (**a**) NMR and (**b**) LC-MS example of the positive ionization mode. Blue circles correspond to the samples incubated in the presence of H_2_O_2_ and extracted at 50 min; red circles correspond to the samples incubated in the absence of H_2_O_2_ and extracted at 50 min; green circles correspond to the samples extracted at 24 hours with and without H_2_O_2_.

**Figure 3:**
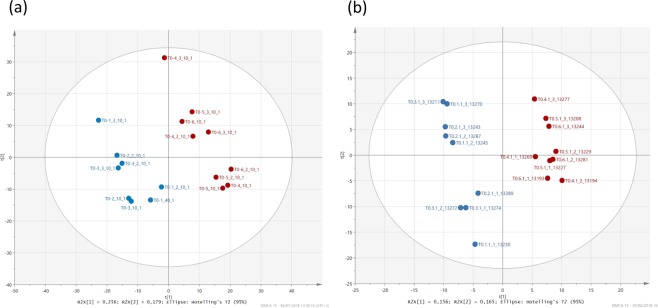
PLS-DA analyses. Score plots (UV scaling) obtained for bacteria metabolomes extracted at 50 min of incubation in the presence (blue circles) or in the absence (red circles) of H_2_O_2_: (**a**) NMR and (**b**) LC-MS (example of the positive ionization mode).

First, statistical analyses (PCA, Figs S1 and S2; PLS-DA, Figs 2 and 3) were done without any preconceptions on the dataset. Therefore, all samples i.e., extracted at 50 min and 24 h incubated in the presence/absence of H_2_O_2_, respectively, were considered, in order to highlight potential discriminations between the samples and to identify which compounds caused these significant differences. PLS-DA analyses were performed on LC-MS and NMR data from (i) cells incubated with H_2_O_2_ at 50 min, (ii) cells incubated without H_2_O_2_ at 50 min, (iii) cells incubated with H_2_O_2_ after 24 h of incubation and (iv) cells incubated without H_2_O_2_ at 24 h. Three classes are observed where samples at 24 h are grouped in a single class (Fig. 2); the model is valid and robust. In addition, a univariate statistical test (Wilcoxon-Mann-Whitney) based on relative intensities of ions and buckets for LC-MS and NMR analyses was carried out to confirm this grouping of samples at 24 h. At 50 min, most of buckets or ions were statistically different reflecting a majority of p-values strictly inferior to 0.05. The situation at 24 h was completely different, and the majority of data were not discriminating with p-value strictly greater than 0.05 (Fig. S3).

#### Identification of key metabolites, markers of H_2_O_2_ exposure

Based on statistical analyses, it is clear that the metabolome of cells incubated with or without H_2_O_2_ were different at time 50 min, while there is no significant difference at time 24 h between the two groups of cells (exposed or not to the oxidant). To highlight the key metabolites involved in the response to the presence of hydrogen peroxide, we focused our study on the sample extracted at 50 min. In order to identify key markers of H_2_O_2_ exposure, PLS-DA was done on data from samples collected at 50 min, incubated or not in the presence of H_2_O_2_. A clear discrimination was observed between the two groups confirming that H_2_O_2_ had a significant effect on bacteria (Figs 3 and S4). Finally, using UV scaling lists of VIP scores 338 discriminating buckets (NMR), 119 and 79 discriminating ions (LC-MS, positive and negative mode) (VIP value >1) were obtained. In addition to the selection based on the value of VIP score, a second selection was done based on the results of the Wilcoxon Mann Whitney test (p-value < 0.05). Finally, 229 buckets, 93 (positive mode) and 76 (negative mode) ions were kept for the identification step. Table 1 summarizes the 60 VIP identified metabolites. Ratios S (Stressed: in the presence of H_2_O_2_)/R (Reference: in the absence of H_2_O_2_) were calculated for each compound based on relative intensities (ions and/or buckets) to evaluate if the compound was more/less present in the samples with H_2_O_2_.

**Table 1:** Metabolites identified according to LC-MS and NMR analyses.

Identified VIP metabolite	NMR		LC-MS			ID metrics (class)
	VIP Score	Ratio S/R	Ionization mode	VIP Score	Ratio S/R	
**Amino acids**						
Isoleucine	1.82–1.89	1.61–1.67	Pos	2.40	2.47	II (9)
Leucine	1.11–1.43	1.23–1.32				
Valine	1.93–2.10	4.35–4.55	- Neg	− 2.15	4.99–5.26	I (9)
Lysine	1.12–1.03	1.12–1.13				(9)
Arginine	1.16–1.49	1.16–1.47				(7)
Proline	1.23–1.49	0.75–0.63				(3)
Glutamate	1.43–1.39	0.68–0.71	Pos - Neg	1.12–1.48	0.72–0.70	I (5)
Methionine	1.33	1.22	Pos - Neg	1.83–1.60	1.41–1.52	I (9)
Alanine	1.81	0.88	Pos	1.33	0.72	II (9)
Beta-Alanine	1.10	1.41				(5)
Aspartate	1.03–1.13	1.11–1.16				(9)
Asparagine	1.21	0.82				(5)
Phenylalanine			Pos - Neg	2.57–2.13	2.29–2.08	I
Tyrosine	1.40–1.53	1.44–1.52	Neg	1.58	2.10	I (9)
Glycine	Detected but not VIP		Pos	1.73	1.38	I (9)
Citrulline	1.01–2.00	0.84–0.81				(5)
Tryptophan	2.17	6.98				(5)
**Saccharides**						
Trehalose*	1.74–1.69	0.85–0.85	Pos	1.96	0.74	II (9)
Trehalose 6-P*			Pos - Neg	2.27–2.04	0.52–0.54	II (9)
Cellobiose*	1.82–1.01	1.26–1.21	Pos	1.96	0.74	II (9)
Cellobiose 6-P*			Pos - Neg	2.27–2.04	0.52–0.54	II (9)
Glycogen	1.80–1.76	0.65–0.84				(9)
Alpha-glucose	1.71–1.69	1.52–2.19				(9)
Fructose	1.15–1.22	1.59–1.79				(2)
Maltose	1.40	1.65				(2)
Xylose			Neg	1.54	0.45	II (9)
**Amines**						
Putrescine	1.16–1.23	1.32–1.16				(7)
Carnitine	1.16	1.06	Pos	1.48	1.76	I (9)
Spermidine			Pos	1.04	1.46	I
**Lipids**						
Palmitoleic acid			Pos - Neg	1.77–1.52	0.36–0.41	I
Lyso PE (16:1)			Pos - Neg	2.07–1.71	0.44–0.50	II (3)
Lyso PC (16:1)			Pos	1.59 -	0.65	II (3)
Lyso PE (16:0)			Pos - Neg	1.97–1.72	0.41–0.36	I
Lyso PC (16 :0)			Pos	1.73	0.41	II (3)
Lyso PE (18:1)			Pos - Neg	2.07–1.55	0.40–0.39	I
**Miscellaneous**						
Acetate	1.01	0.84				(6)
Lactate	1.09	1.41				(9)
Succinate	1.05	1.40				(6)
Citrate	1.33–1.28	1.15–1.22				(3)
Beta-hydroxybutyrate	1.59–1.44	1.25–1.26				(5)
Pipecolic acid	1.96–1.59	1.46–1.25				(4)
Pantothenic acid	1.05–1.02	1.40–1.21	Pos - Neg	2.31–1.76	1.90–1.92	I (9)
Inosinic acid	1.73–1.41	0.71–0.72				(9)
Nicotinamide ribotide	1.19–1.03	0.86				(3)
AMP	1.73–1.38	0.72–0.76				(9)
ATP	2.09–2.17	0.43–0.40				(9)
NADH	1.37–1.38	0.75–0.76				(2)
UMP	2.04–1.92	0.43–0.45	Neg	2.03	0.47	(9)
Glycerol	1.03	0.90				(9)
Glycerol 3-P			Neg	1.65	0.49	I
Betaine	2.13	4.46	Pos	2.70	5.14	I (2)
Glutathione	1.08	1.28				(9)
**Peptides**						
Alanyl-alanine			Pos	2.01	0.65	I
Isoleucyl/leucyl-histidine			Pos	1.99	2.35	II (3)
Isoleucyl/leucyl-aspartic acid			Pos	2.58	6.14	II (3)
Valyl-isoleucine/leucine			Pos	1.74	4.89	II (3)
Valyl-serine			Pos	1.17	0.71	I
Valyl-aspartic acid			Pos	2.00	1.95	I
Gln-Asp-Thr-Pro			Pos	2.32	4.31	II (3)
Arg-Cys-Ser-Trp			Pos	1.85	0.57	II (3)

VIP scores indicate the relative influence of the corresponding metabolites on the discrimination between the two conditions (with and without H_2_O_2_) for both analytical techniques. Ratios S (in the presence of H_2_O_2_)/R (in the absence of H_2_O_2_) were determined from relative intensities in profiles at 50 min. Classes and scores are according to Sumner *et al*.^59,60^.

*Under our analytical conditions, Trehalose/Cellobiose and Trehalose 6-P/Cellobiose 6-P were not differentiated.

The use of two different and complementary analytical techniques (NMR and LC-MS, see Tables S1, S2, S3) allowed detecting various metabolites, which are key markers of the response of *P*. *graminis* 13b-3 to hydrogen peroxide stress (Table 1): amino-acids, saccharides, amines, lipids, peptides and miscellaneous compounds. NMR analysis allowed detecting most of “miscellaneous” metabolites such as AMP, ATP, NADH, betaine, acetate, lactate, succinate, citrate, β-hydroxybutyrate, pipecolic acid, inosinic acid, nicotinamide ribotide, most sugars and also glutathione. LC-MS allowed the detection of lipids, some amino acids, peptides and others compounds such as glycerol-3P, spermidine and xylose. Some metabolites were detected with both NMR and LC-MS analyses. It is the case for amino-acids (leucine, valine, alanine, methionine, tyrosine, and glutamate), saccharide (trehalose) and miscellaneous compounds (pantothenate and UMP).

## Discussion

Our results clearly show the metabolic response of *Pseudomonas graminis* 13b-3 to oxidative conditions such as existing in clouds.

**Figure 4:**
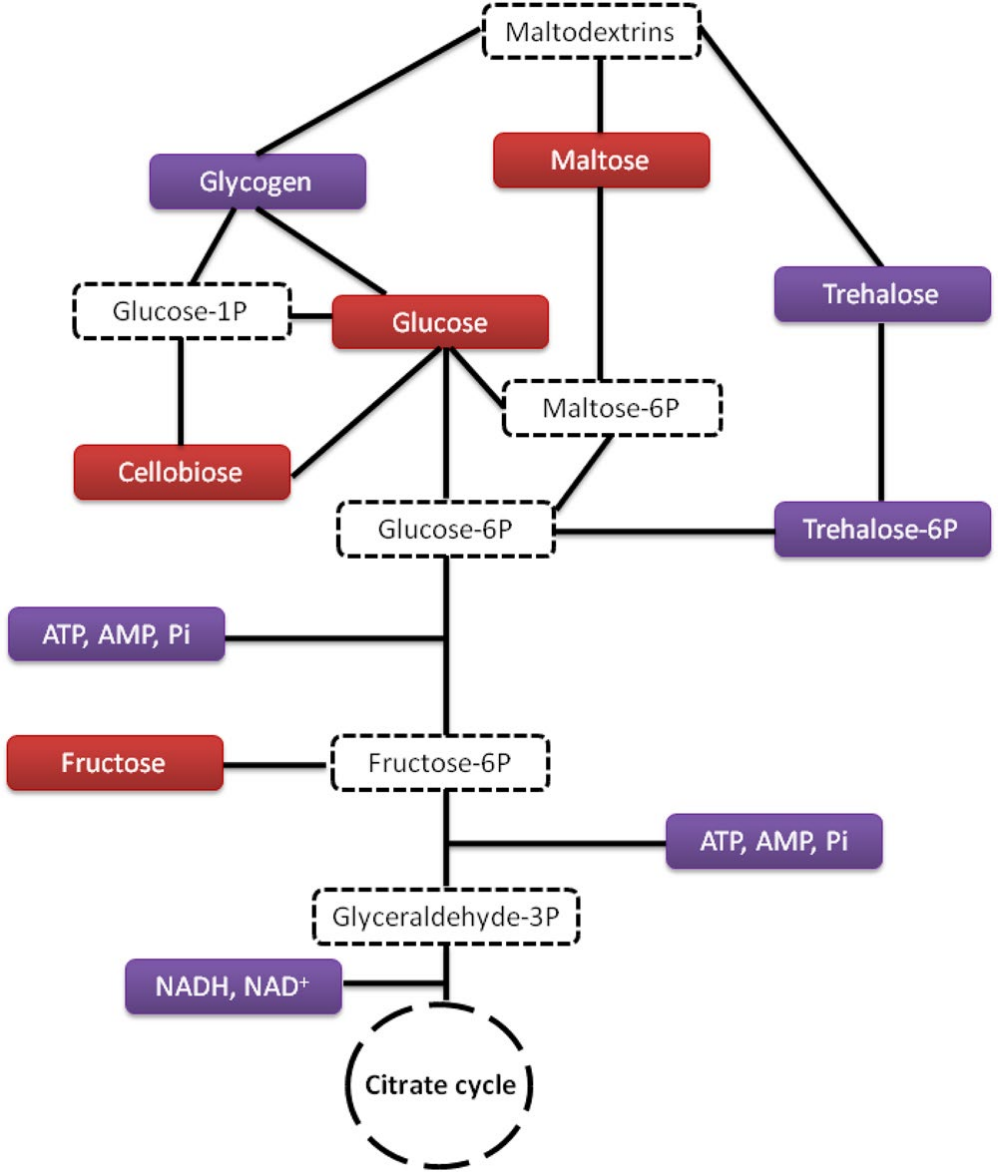
Carbohydrate metabolism showing metabolites over-produced in the presence of hydrogen peroxide (red boxes) and those under-produced (purple boxes). Likely metabolic intermediates are indicated in white boxes with dotted frame.

**Figure 5:**
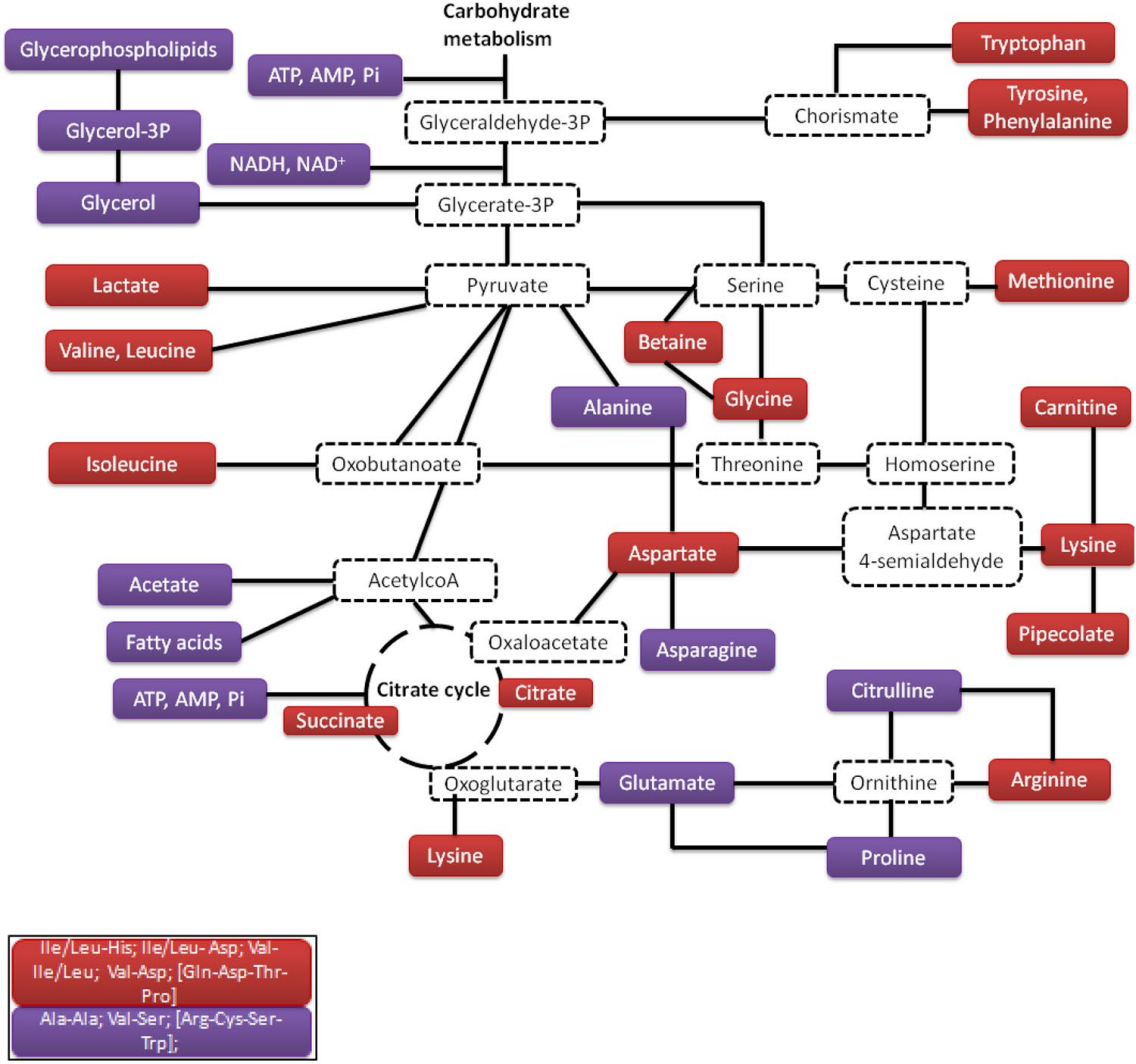
Central metabolism: Citrate cycle, lipids, amino acids and peptides. The metabolites, which are over-produced in the presence of hydrogen peroxide, are indicated in red boxes, while those which are under-produced are indicated in purple boxes. Putative metabolic intermediates are indicated in white boxes with dotted frame.

**Figure 6:**
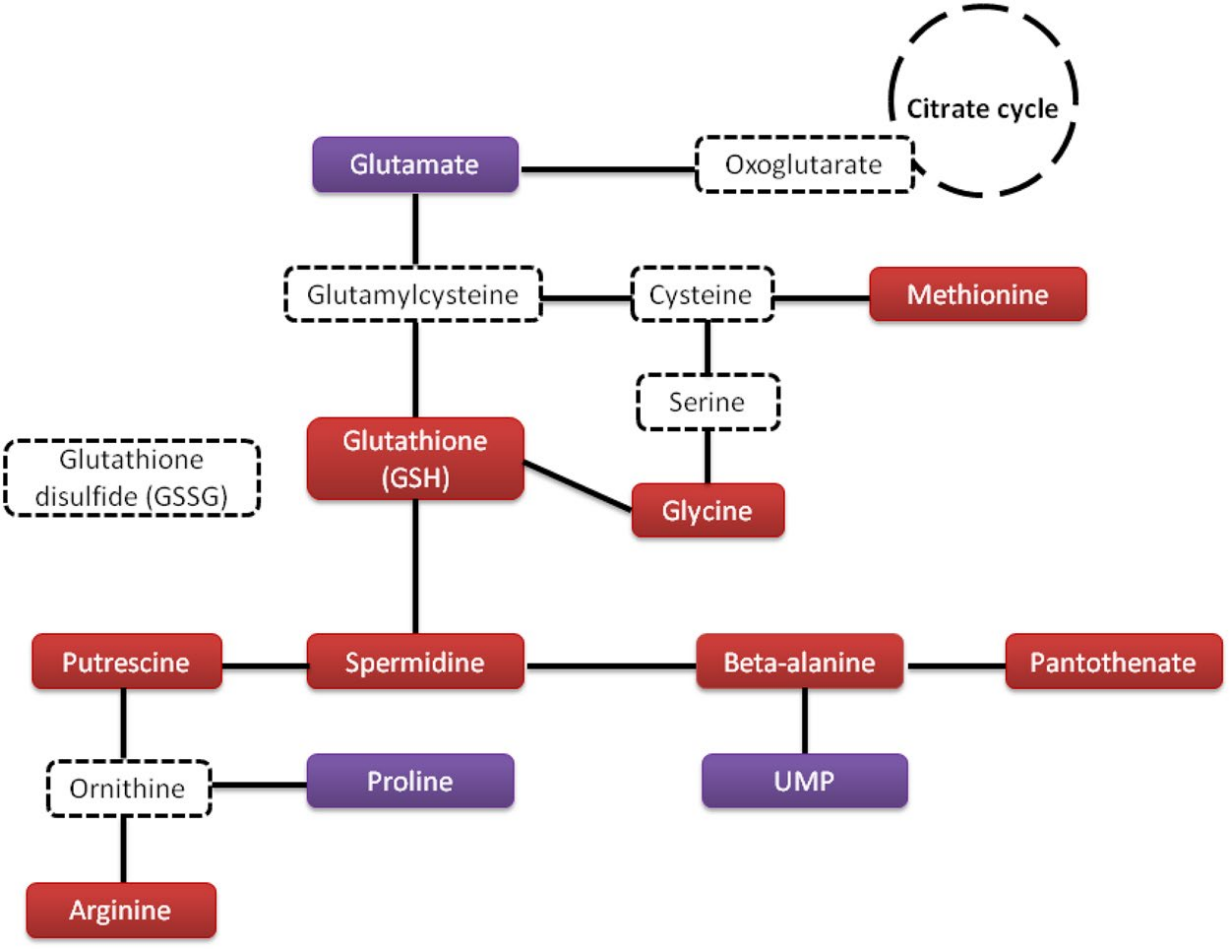
Glutathione metabolism. The metabolites, which are over-produced in the presence of hydrogen peroxide are indicated in red boxes, while those, which are underproduced, are indicated in purple boxes. Putative metabolic intermediates are indicated in white boxes with dotted frame.

First, metabolic pathways of *Pseudomonas graminis* are highly impacted under hydrogen peroxide stress. These impacted pathways (Figs 4–6) were mapped using Kegg data base along with the strain’s genome annotation^32,33^. Carbohydrates pathway is displayed in Fig. 4. Carbohydrates are important sources of energy in living cells; their metabolism was highly impacted by the presence of H_2_O_2_. This metabolism is complex showing interconversion of various poly-, oligo- and di- and mono-saccharides. Glycogen and trehalose (as well as its derivative trehalose-6 phosphate) concentrations were decreased in the presence of H_2_O_2_, while those of maltose, glucose, cellobiose and fructose were increased. These metabolic changes showed that this bacterium accelerates the biodegradation of its storage compounds (glycogen and trehalose) into maltose and glucose. Glucose and maltose entered the glycolysis and formed fructose from fructose 6-phosphate.

Soluble sugars, especially glucose and fructose, were involved in the responses to a number of stresses including oxidative stress due to the presence of ROS. It has been suggested earlier that sugar-signalling and sugar-modulated gene expressions are involved in the control of oxidative stress expression^34^.

Carboxylic acid concentrations were modified in the presence of H_2_O_2_: lactate, citrate and succinate were found at higher amounts while acetate concentration is decreased (Fig. 5). This result is consistent from that observed in *E*. *coli* exposed to H_2_O_2_ where fumaric acid, succinic and malic acids are also more abundant^30^.

Three classes of lipids were detected by LC-MS analysis (palmitoleic acid, three lyso PE (lyso-phosphoryl-ethanolamine) and two lyso PC (lyso-phosphoryl-choline)). Palmitoleic acid is part of fatty acid metabolism, while lyso PC and lyso PE are part of glycerophospholipid metabolism (Fig. 5). Except for one type of lyso PC, all the VIP scores were high (average around 1.80), so lipids were strongly discriminating metabolites in the expression of oxidative stress. In addition, the low values of S/R ratios measured at 50 min (average around 0.40) for all lipids indicated that these metabolite concentrations were lower in the presence of hydrogen peroxide (Table 1). In parallel, the concentration of glycerol and glycerol-3-phosphate, two intermediates of the phospholipid metabolism, were decreased under oxidative conditions. This observation is consistent with the fact that lipids are major targets of oxidative stress. Free radicals can attack directly polyunsaturated fatty acids in membranes and initiate lipid peroxidation. A primary effect of lipid peroxidation is a decrease in membrane fluidity, which alters membrane properties and can disrupt membrane-bound proteins significantly^35^. Membrane damage disrupts cellular integrity, causing loss of the proton motive force that drives ATP formation and the uptake of many nutrients^36^. However, further studies should be conducted in the future to explore in more detail lipid metabolism using different extraction and HPLC conditions. These will give more information about the oxidative state of lipids under oxidative conditions.

Energy metabolism was strongly impacted by the presence of hydrogen peroxide; at 50 min, when bacteria wzre exposed to the oxidant, the concentrations of adenosine, monophosphate (AMP) and adenosine triphosphate (ATP) were decreased (Table 1, Fig. 5) as well as that of inosinic acid, an intermediate of ATP and AMP synthesis. This observation is consistent with the results shown in Fig. 1 and also in Wirgot *et al*.^11^. Indeed, at 50 min the ATP concentration was about five times lower in the sample incubated with hydrogen peroxide than in the reference. The present study showed a decrease of 2.5-fold (S/R = 0.4). ATP is listed among the highest VIP metabolites (2.1 score). A few studies also reported a decrease of ATP concentrations in cells exposed to H_2_O_2_; these include *Streptomyces pneumoniae*^37^ and cultured mammalian and plant cells^38–40^.

NADH concentration was also decreased in the presence of H_2_O_2_, which could result from a tuning of the cell redox potential (NAD^+^/NADH, NADP^+^/NADPH ratios). As H_2_O_2_ impacts glutathione metabolism, glycolysis, TCA cycle and DNA repair system, it also indirectly impacted these NAD^+^/NADH, NADP^+^/NADPH co-enzymes^11,41,42^. NADH decrease could also have resulted from modulations for rather favoring the production of NADPH, involved in many anti-oxidant enzymatic reactions, at the expense of NADH as reported in *Pseudomonas fluorescens*^31,42^.

A large number of amino-acids were identified as VIP metabolites in this study (Table 1, Fig. 5). The concentrations of 75% of the amino-acids detected (isoleucine, leucine, valine, lysine, arginine, methionine, aspartate and cyclic compounds like tryptophan, phenylalanine, and tyrosine) were increased at 50 min under oxidative stress. S/R ratios of valine and tryptophan were particularly high (4.5 and 6.98 respectively) compared to the others with a high VIP score (around 2.0) suggesting that these could be strong key markers of the response of *P*. *graminis* to H_2_O_2_ stress. Some compounds, which are connected to amino acid metabolism, were also overproduced, for instance carnitine and pipecolate linked to lysine metabolism or betaine linked to glycine metabolism. Betaine concentration was particularly increased (S/R = 4.46) and presented a high VIP value (2.13). This metabolite is also found in the case of cold shock^43^. However, some amino acid concentrations were decreased in the presence of H_2_O_2_, including alanine, glutamate, citrulline, proline and asparagine. Jozefczuk *et al*.^30^ also observed an overproduction of most of the amino acids in *E*. *coli* exposed to H_2_O_2_, although some differences can be noticed concerning the exact nature of these amino acids (for instance alanine is overproduced in *E*.*coli* while it is underproduced in our case).

Some amino acids (glutamate, glycine) or amines linked to amino-acid pathways (carnitine, betaine) are well known osmo- or cryo-protectants. These compatible solutes accumulate in the cells facing osmotic or cold shocks. Carnitine is known as an antioxidant compound^44^. Except for glutamate whose concentration was decreasing, glycine, carnitine and betaine were accumulating in *P*. *graminis* 13b-3 in the presence of H_2_O_2_. This is consistent with studies that report a concomitant response to oxidative stress and cold shock^45^.

Interestingly, the modifications of amino acid concentrations were accompanied by the modulation of dipeptide and tetrapeptide concentrations (Table 1, Fig. 5). Ala-Ala, Val-Ser and [Arg-Cys-ser-tyrp] concentrations decreased under stress conditions while the concentration of Ile(leu)-His, ILe(leu)-Asp, Val-Ile(leu), Val-Asp and [Gln-Asp-Thr-Pro] increased. It is worth noting that some of these peptides had the highest VIP and S/R values of all the metabolites of interest. It is particularly true for ILe(leu)-Asp (VIP score = 2.58, S/R = 6.14) and [Gln-Asp-Thr-Pro] (VIP score = 2.32, S/R = 4.31). These peptides seem, thus, of major interest and might be the more important key markers of H_2_O_2_ stress for *Pseudomonas graminis* 13b-3. The discovery of dipeptides and tetrapeptides was unexpected as they have never been described as a response to oxidative stress. Only a very few previous studies report the presence of short peptides in bacterial cells. Kol *et al*.^46^ reported an overproduction of one dipeptide (Gly-Pro) and four tripeptides (Arg-Gly-Pro, Lys-Gly-Pro, Glu-Gly-Pro and Ala-Gly-Pro) in response to a salt shock in *Steptomyces coelicolor* based on a metabolomics approach. These authors suggested that these peptides all containing proline could be hydrolyzed by the cell to produce proline, which is a known osmoprotectant. The overproduction of these peptides was concomitant with the increase of proline concentrations. The presence of proline-containing short peptides was also described earlier in *Listeria monocytogenes*^47^, *Lactobacillus casei*^48^ *Oenococcus oeni*^49^ and *Bacillus subtilis*^50^ facing osmotic shocks. In our case, only one tetrapeptide [Gln-Asp-Thr-Pro] contained proline and - although it was overproduced in the presence of H_2_O_2_ - proline concentration was not increased but decreased. The mechanism involved there seems thus quite different, and the exact role of these dipeptides and tetrapetides remains unknown. Using a metabolomics approach, Jousse *et al*.^43^ observed the presence of short peptides in *Pseudomonas syringae* 32b-74 under cold shock. In this latter case, these peptides were not specifically containing proline and they were not all overproduced. In parallel, the free amino-acid concentrations were mainly decreased under cold conditions. It was suggested that short peptides could play unknown roles in regulating pathways. In the present study, the modulation of dipeptide and tetrapeptide concentrations could be also related to regulating pathways. In addition, when peptides were accumulating, we cannot exclude their potential role in the protection against oxidative damages. For instance, it was shown earlier that linear glycine-containing dipeptides could scavenge radicals in human erythrocytes when exposed to oxidative stress^51^.

As expected during oxidative stress, glutathione GSH concentration increased in *P*. *graminis* (Fig. 6, Table 1). However, we can note that glutathione was not among the highest VIP metabolites (1.08 score) and the S/R ratio was not very high (1.28). Glutathione is widely found in many living organisms including microorganisms; it has various functions involved in the defense against oxidative stress^11,44^. Consistently with the increase of glutathione concentration, other metabolites directly linked to the same pathway, such as glycine, methionine, putrescine, arginine, spermidine, beta-alanine, UMP and pantothenate, were also foun increased in the presence of oxidants in our study (Fig. 6).

Putrescine and spermidine are among the most widely distributed cellular polyamines and these are essential for normal cellular growth and multiplication of both prokaryotic and eukaryotic cells. Spermine and spermidine can act as free radical scavengers and reduce cellular damages induced by oxygen radicals^52^. Polyamines therefore play an important adjunctive role in protecting cells from the toxic effects of ROS.

This work shows of the plasticity of *P*. *graminis* 13b-3 metabolome in clouds. *Pseudomonas graminis* responded to oxidative stress by modifying many metabolic pathways (carbohydrates, lipids, amino-acids, amines, glutathione, peptides). However, *P*. *graminis* was short after able to recover, as attested by increasing ATP concentration (Fig. 1) and using similar metabolic pathways as the reference cells (Fig. 2) when the medium had been completely detoxified. These results clearly demonstrate the flexibility of *P*. *graminis* after H_2_O_2_ exposure. This is consistent with the fact that this bacterium and others isolated from clouds survive when exposed to H_2_O_2_ at high concentrations and were even able to grow after H_2_O_2_ exposure^10^. Previous experiments also showed a very strong correlation between ATP and cloud H_2_O_2_ concentrations measured directly in cloud water^11^. This suggests a great plasticity of the cloud microorganism metabolome which constantly adapts to the H_2_O_2_ concentrations. This aspect is particularly important in the atmospheric context as H_2_O_2_ concentration is highly variable in clouds depending on various parameters (e.g. photolytic activity, temperature and concentrations of trace gases, such as VOCs, CO, O_3_, and NO_x_). Sunlight is particularly important; as a result, diurnal and seasonal variations of H_2_O_2_ concentration are observed. In the long term, the atmospheric concentration of H_2_O_2_ might increase due to the increasing concentration of pollutants such as NO_x_^2^.

The very high oxidative condition present in clouds (up to 200 mV) is probably one of the major constraints experienced by cloud microorganisms. Consistently, a recent metatrancriptomics analysis of the cloud microbiome highlighted in particular the expression of genes involved in oxidative stress response, antioxidant activity, as well as glutathione biosynthesis in bacteria^53^.

Finally our work points out the interactions between the bacterial metabolome and cloud chemistry.

**Figure 7:**
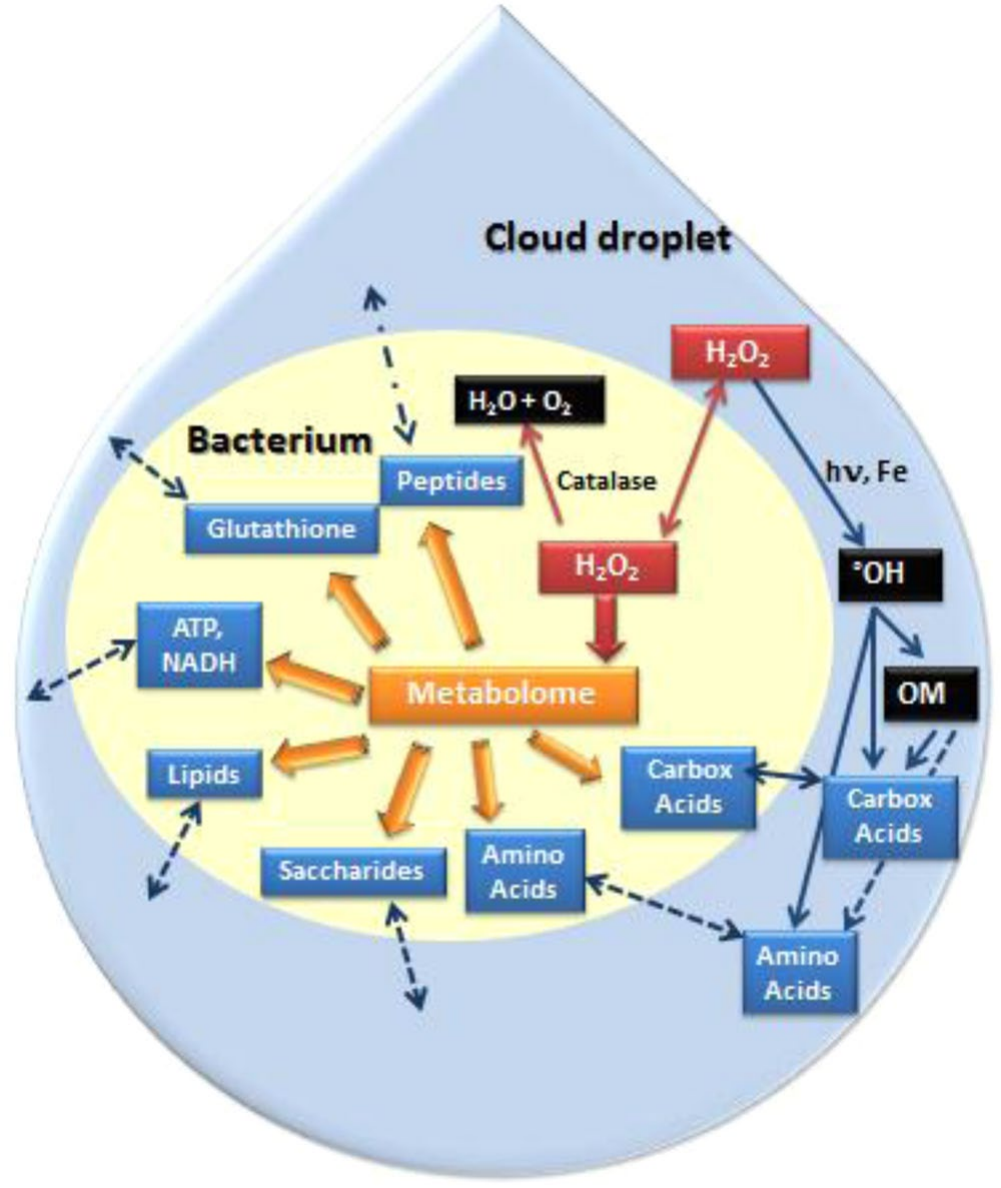
Simplified scheme of the interactions of H_2_O_2_ with the bacterium metabolome and the organic matter (OM) present in the cloud droplet. The modulation of the metabolome by H_2_O_2_, in particular, amino acid and carboxylic acid pathways, could have an impact on cloud chemistry by changing the amino acid and carboxylic acid concentrations in the cloud droplet. Carbox acid: carboxylic acids. Dotted lines indicate unknown pathways (transport, reactions…).

Hydrogen peroxide is a key component in the chemical reactivity in the atmosphere, driving most of the radical reactions as a major source of hydroxyl radicals (°OH)^2^ (Fig. 7). In the water phase of clouds, H_2_O_2_ can interact with iron and/or UV light to produce °OH radicals by Fenton, photo-Fenton or direct photolysis reactions, even though the major part of °OH radical is likely transferred from the gas phase to the water phase of clouds. OH is the main oxidant for organics in cloud water^3^. This OM is very complex, and its molecular composition remains still unknown. Two major classes of small organic compounds have been identified and quantified, namely carboxylic acids and aldehydes (10%) and amino acids (9%)^1,4,5^. These compounds are also major components of fog and aerosols^4,7^ and contribute to atmospheric chemistry and the global carbon and nitrogen cycles. The origins of carboxylic acids are direct emission, transformation in the gas phase and transfer to the cloud droplet, or they can also result from the transformation of the OM by radical chemistry directly in cloud water^4^. The origin of amino acids is not clearly established as they have been discovered only recently in clouds^5^. Free amino acids, peptides and proteins have been found in aerosols and are likely issued from biological aerosols particles (BAPs)^7^ The presence of “peptide-like compounds” has been shown in cloud water by global FT-ICR-MS analyses although their concentration has not been quantified^8,9^. Both amino acids and carboxylic acids can interact with °OH radicals in the water phase of clouds for further reactions^3–5^.

It was shown that microbial metabolism could be an alternative route to radical chemistry in the transformation pathways of carbon compounds and oxidants in clouds^15,27^. Ariya *et al*.^20^ showed that dicarboxylic acids (C_2_–C_9_), namely oxalic, malonic, succinic, glutaric, adipic, pimelic, and pinic acids could be degraded efficiently by airborne-microorganisms. Using microcosms mimicking the cloud environment or real cloud samples, Vaitilingom *et al*.^19,22,23^ and Husarova *et al*.^24^ showed that the biodegradation rates of organic acids (acetate, succinate, malonate) and of C1 compounds (formate, formaldehyde, methanol) were within the same concentration range as photodegradation rates. In addition, microorganisms isolated from cloud water have been shown to produce organic compounds, such as pyruvate from lactic acid^21^. The biotransformation of amino acids present in clouds has not been published yet, but it is very likely to occur as it was shown that microorganisms can grow using the carbon and nitrogen sources present in cloud water, thus building proteins from amino acids^21^. In addition, the presence of transcripts of genes involved in amino acid pathways has been shown recently in clouds^53^. We have also confirmed here that H_2_O_2_ can be transformed by catalases, consequently microorganism activity could decrease the efficiency of radical chemistry because it reduces the source of radicals^11,19^.

In this work, we have clearly shown that H_2_O_2_ directly impacts the metabolome of a cloud bacterium (Fig. 7). Many pathways were modified including glutathione, saccharide, lipids, energy (ATP, NADH), peptides, amino acids and carboxylic acids pathways. The metabolites of these pathways could be taken up or excreted by the bacterium in the cloud droplet, and thus potentially change the cloud OM composition. This is particularly true for amino acids and carboxylic acids, which are major components of the cloud OM (Fig. 7). Lactate, succinate and citrate were overproduced while acetate was underproduced in the presence of H_2_O_2_. Among the amino acids whose concentrations are modulated under oxidative stress, valine and tryptophan concentrations were drastically increased (around 4.5- to 7-fold). Peptides are also key markers of H_2_O_2_ stress and could change the “peptide-like” concentrations in cloud water. We have shown that *P*. *graminis* has a great metabolome plasticity and the capacity of acclimate to a range of H_2_O_2_ concentrations. This is quite important regarding atmospheric context as H_2_O_2_ concentrations are highly variable. This means that not only cloud microorganisms can compete with radical processes but this competition could be modulated by H_2_O_2_ concentration, i.e. by atmospheric scenarios. We are not able to quantify this modulation at the moment; future work is needed, notably looking at amino acids and peptide transformations. Although this work was based on a single strain, we can expect that this phenomenon is true for all cloud metabolomes as we previously showed a very strong correlation between ATP and H_2_O_2_ concentration measured in real clouds, ATP being a proxy of the whole cell metabolism^11^.

## Material and Methods

The general workflow describing the experiment protocol used for the metabolomics experiments is presented in Fig. S5.

Three independent experiments were performed (three batches of twelve samples each). Furthermore, the biodegradation of hydrogen peroxide as well as the evolution of ATP concentration were followed for each experiment over time. These experiments were conducted in marine artificial cloud solution as the majority of clouds sampled at the puy de Dôme station are from marine origin^23^. The chosen incubation temperature was 17 °C, because it is the average temperature in summer at the puy de Dôme station (1465 m).

In nature, microorganisms are aerosolized and then condense water to become part of the cloud water system, therefore they are directly transferred from their original rich environment (soil, vegetation, surface waters) to the cloud medium containing H_2_O_2_ and have no time to grow first in this new medium before being exposed to H_2_O_2_. This is the reason why we grew the cells in R2A, washed them twice with the cloud medium to eliminate the nutrients and salts (we checked by NMR by there was no nutrient left originating from R2A such as glucose), and transferred them directly in cloud water with or without H_2_O_2_ to really see the impact of H_2_O_2_ on their metabolism.

### Chemical reagents

Hydrogen peroxide solution was freshly prepared just before each experiment from a commercial solution (H_2_O_2_; 30%; not stabilized; Fluka Analytical). Acetonitrile (CH_3_CN; 99.99%; Optima LC-MS; Fisher Scientific), methanol (CH_3_OH; 99.9%; Fluka Analytical) and water (H_2_O; Optima LC-MS; Fisher Scientific) were used in the proportions 2:2:1, respectively, for metabolites’ extraction. Water (H_2_O; Biosolve) and acetonitrile (CH_3_CN; 99.97%) in the proportions 1:1 (v/v), acidified at 0.1% with a formic acid solution (HCOOH; 99%; Biosolve), were used for LC mobile phase. Deuterium oxide (D_2_O; 99.96%D; Eurisotop), phosphate buffer (KH_2_PO_4_; 99.50% & K_2_HPO_4_; 99.50%, at a final concentration of 30 mM; Sigma Aldrich) and sodium azide (NaN_3_; 99.99%; Sigma Aldrich) were used for NMR sample preparation. Carbon source, nitrogen compounds and salts used for artificial cloud solution are commercially available (Table S4).

Standard solutions used for LC/MS validation which were colchicine, tryptophan, phenylalanine, amino anthracene and creatinine were prepared from stock solution (0.5 g/L for each compound in water/acetonitrile 1:1 (v/v) solution containing 0.1% formic acid and stored at −20 °C) diluted in the same solvent at a final concentration of 5.0 10^−3^ g/L before use.

### Basic physiological characteristics of *Pseudomonas graminis* PDD-13b-3

The bacterial strain PDD-13b-3 (GenBank DQ512786) was isolated from cloud water collected on the 7^th^ of August 2004 by impaction from puy de Dôme summit (1465 m asl; France), by culture on R2A medium at 17 °C; it was identified as a species of *Pseudomonas graminis*^17^. Its draft genome was sequenced recently and is available under the Bioproject accession number PRJNA362581^32^. In the laboratory, this bacterium is able to grow at temperatures at 5 °C, 17 °C, 27 °C and 37 °C (lower and higher temperatures were not investigated) in R2 medium, with generation times of 8.6, 4.45 and 3.7 hours at 5 °C, 17 °C and 37 °C respectively, and an optimum for growth at 27 °C with 1.2 h/generation”. Its cellular ATP content measured during exponential growth in R2 at 17 °C is 5.5 ( ± 0.7) 10^−18^ mol cell^−1^. Previous work showed that this strain is able to uptake and use as unique C sources acetate, formate, L-lactate, succinate, formaldehyde, and to a lesser extent also methanol^21^.

### Microbial cultivation and incubations in microcosms

For this work, bacteria were grown aerobically in 100 mL of R2A medium under agitation (200 rpm) at 17 °C for 17 hours. Then, cells in the exponential growth phase were collected by centrifugation for 3 min at 10,500 g. The supernatant was removed, and the bacterial pellet was suspended and washed twice in artificial cloud solution^23^. The concentration of cells was estimated by optical density measurement at 575 nm and diluted to obtain a concentration of ~10^7^ cell.mL^−1^; biomass was further precisely quantified by flow cytometry (BD Facscalibur Becton-Dickinson; λ_exc_ = 488 nm; λ_em_ = 530 nm) on SYBR green stained samples. The artificial cloud solution was used to mimic cloud conditions; it contains the major carbon and nitrogen compounds and salts present in clouds originating from the ocean when samples are collected at the sampling site (Table S4). H_2_O_2_ concentration was fixed at 200 µM for our experiments. The cell concentration/chemical concentration ratio (Carbon compounds, ions and oxidants) was kept identical to that of a real cloud. We showed that biodegradation rates are kept constant when the cell/substrate ratios are constant^22^.

Amber Erlenmeyer’s flasks containing 150 mL of cloud water solution were inoculated with 10^7^ cells.mL^−1^ and incubated at 17 °C, 110 rpm, and half were added with 200 µM of hydrogen peroxide. Two Erlenmeyer flasks (with and without H_2_O_2_) were used to monitor the evolution of hydrogen peroxide and ATP concentrations over time. The experiment was repeated three times, resulting in three biological replicates. Hydrogen peroxide concentration was measured by fluorescence spectroscopy (Safire II TECAN^©^; λ_exc_ = 320 nm, λ_em_ = 390 nm) as in Wirgot *et al*.^11^. This method is based on a reaction between hydrogen peroxide, horse radish peroxidase and p-hydroxyphenylacetic acid. Bioluminescence was used to determine ATP concentration (Glomax® 20/20 single tube luminometer from Promega); this is based on the endergonic oxidation of D-luciferin by the enzyme luciferase, and producing an amount of photons directly proportional to that of ATP in the sample^11^. The protocol used was adapted from ATP Biomass Kit HS by Biothema (Sweden).

Twelve Erlenmeyer flasks were used to study the modulation of the bacterial metabolome in the presence or absence of H_2_O_2_. After 50 minutes and 24 hours of incubation (i.e., time after H_2_O_2_ addition), respectively, subsamples were collected, cell pellets were obtained by centrifugation (12000 g, 4 min, 4 °C) and rinsed twice in 0.8% sodium chloride solution (w/v).

Centrifugation followed by a quick quenching with cold solvent was chosen instead of filtration because it was more adapted to the high volumes of samples (12 samples of 150 mL). The advantage of centrifugation is that all the samples are centrifuged simultaneously. A simultaneous treatment is important because the incubation is stopped at 50 min, therefore any additional minutes between the samples due to extraction/quenching procedure will lead to very heterogeneous results.

### Samples preparation for metabolomics

The metabolomes were extracted from pellets using 1.2 mL of water/methanol/acetonitrile [1:2:2] solution kept in an ice bath^43^. After a last step of centrifugation intended to remove cell debris (5 min, 4 °C, 12000 *g*), 750 µL of the supernatants were dedicated to NMR and four aliquots of 50 µL were dedicated to LC-MS analyses. Moreover, five aliquots of 10 µL of each sample were mixed together for serving as quality controls through LC-MS analyses.

For NMR analyses, the supernatants were evaporated at 10 °C for approximately 48 hours using a refrigerated centrivap concentrator equipped with a centrivap cold trap at −50 °C (LabConco; ThermoScientific). The extracts were suspended into 300 µL of deuterium oxide (D_2_O, 99.96%D) and dried two times in order to eliminate H_2_O traces. Finally, the extracts were suspended in 90% of phosphate buffer (30 mM) prepared in D_2_O (99.96%D).

### Metabolic profiling

#### LC-MS analyses for metabolite profiling

LC-MS analyses were performed on the Metabolic profiler® platform (Bruker) using a fast LC system (Agilent 1200 series) coupled to a MicroToF mass spectrometer equipped with electrospray ionization (ESI) (Bruker). Chromatography separation was performed on an Acquity HSST3 C18 column (150*2.1 mm 1.8 µm; Waters). The flow rate was fixed at 0.2 mL/min with 1 L of water and acetonitrile containing 0.1% of formic acid for mobile phases A and B, respectively. The gradient elution was carried out as follows: 0–2 min, 100% A; 2–15 min linear gradient to 0% A; 15–22 min, 0% A; 22–22.10 min linear gradient back to 100% A; 4 min equilibration wash with 100% A. The injection volumes for all samples were 6 µL and the column temperature was set to 30 °C. The mass spectrometer operated with full scan (50–1000 m/z) in positive and negative ion modes. Nitrogen was used as the drying gas. The gas nebulizer (nitrogen) pressure was 40 psi, the desolvation gas flow rate was 9 L/min and temperature was maintained at 200 °C. Capillary tension was 4500 V. Finally, softwares used for data acquisition were OtofControl 3.2 and Hystar 3.2 (Bruker Daltonics).

Matrix of relative intensities for all samples (Stressed and Reference) corresponding to each identified metabolite obtained for LC-MS analyses is shown in Table S5. Means, standard deviations and ratios S/R were calculated.

#### 1D NMR analyses for metabolite profiling

One-dimensional ^1^H NMR experiments were done with an Avance III 500 MHz NMR spectrometer using a 5 mm Prodigy TCI z-gradient (^1^H/^19^F/^13^C/^15^N) probe (Bruker Biospin Wissenbourg, France). Samples were analyzed on 5 mm tubes refrigerated at 6 °C on an auto-sampler (Sample Jet). NMR spectra were recorded at 25 °C K. A standard one-dimensional *noe* spectroscopy sequence (*noesygppr1d* with water presaturation and gradients) was used with low power irradiation of the water resonance during the recycle delay of 2 s and the mixing time of 10 ms. A total of 1024 scans was collected with a 90° impulsion time of 8.70 μs, acquisition time of 3.3 s, spectral window of 64 K data points zero-filled to 128 K before Fourier transformation with 0.3 Hz line broadening. All spectra were finally processed with Topspin version 3.5pl5 (Bruker Daltonics).

### Data treatments

#### LC/MS preprocessing of chromatograms

MS raw data were processed using the web-based platform Galaxy framework including preprocessing, normalization and quality control steps^54^. XCMS software implanted in the Galaxy platform was used to process raw data for feature detection, alignment and framing^55^. This step produced a table of features characterized by sample retention time, mass to charge (*m/z*) ratio and intensity (i.e., peak area). XCMS parameters used for data processing are shown in Table S6. After we observed an analytical deviation of signals, intensities of peaks were corrected with a regression model in order to remove signal drift and batch-effects^56^. The stability of signal intensities across batches was then evaluated by relative standard deviation (RSD) of each feature in QCs (quality control standard). Features with RSD in QCs higher than in samples were excluded. We kept only features with RSD in QCs below 30% for further multivariate analysis.

#### NMR preprocessing of spectra

1D ^1^H spectra were divided into regions (buckets) of 0.01 ppm width. Over the chemical range of 0.5 (right border) to 10 (left border) ppm, values were not used in the bucketing. Furthermore, the area around residual water signal (4.6 to 5.1 ppm) was excluded. Bucketing was performed using the AMIX software (Bruker GmbH). The signal intensity in each bucket was normalized by the total intensities of spectra. Finally, a data matrix of relative intensities characterized by chemical shifts was obtained and used for multivariate analysis.

Matrix of relative intensities for all samples (Stressed and Reference) corresponding to each identified metabolite obtained for NMR analyses is shown in Table S7. Means, standard deviations and ratios S/R were calculated.

### Statistical analysis

The data matrices obtained for LC-MS (positive and negative mode) and NMR (^1^H) were subjected to multivariate and univariate analyses. First, multivariate analyses were performed using SIMCA-P software (v12, Umetrics) to identify a potential discrimination between the samples incubated in the presence or absence of H_2_O_2_ and for the two times of incubation. Principal Component Analysis (PCA (Figs S1 and S2) and Partial Least Square Discriminant Analysis (PLS-DA, Figs 2 and 3) were done. A selection of discriminant ions and buckets was done based on variable importance in projection (VIP) values. The values strictly greater than unity were kept for the identification step. In addition to multivariate analyses, univariate analysis was done (Wilcoxon-Mann-Whitney test); ions and buckets with a p-value lower than 0.05 were kept for the identification step.

### Identification of discriminants variables

#### UPLC-MS/MS analyses for structural identification of the metabolites

CAMERA R package was used to annotate adduct peaks, isotopes and in-source fragments of compound spectra in positive and negative mode^57^. QCs were reinjected in order to identify the most discriminant features in multivariate analysis. Each feature was identified from in-source fragments and adducts. Additional structural information was obtained by performing MS/MS fragmentation using UPLC-qTOF-MS/MS. MS/MS fragmentation patterns were compared to simulations through Massfrontier (v.7; Thermo Scientific) software. When standards were available, these were analysed to confirm putative identifications.

#### 1D and 2D NMR analyses for structural identification of the metabolites

For better sensitivity and resolution of spectra, a NMR spectrometer *Bruker* Avance III 950 MHz equipped with a 5 mm TCI (^1^H/^13^C/^15^N/^2^H) cryoprobe with z-gradient coil probe (Bruker Biospin Wissenbourg, France) was used, in order to identify discriminant buckets. For 1D ^1^H-spectra, a standard one-dimensional spectroscopy sequence (noesygppr1d) was used with low power irradiation of the water resonance during the recycle delay of 4 s and the mixing time of 10 ms. For each spectrum, eight scans were collected with an 90° impulsion time of 7.7 μs, an acquisition time of 3.3 s, a spectral window of 10 000 Hz and 64 K data points zero-filled to 128 K before Fourier transformation with 0.3 Hz line broadening. All 2D homonuclear (^1^H-^1^H COSY, ^1^H-^1^H TOCSY, ^1^H-^1^H JRES) and heteronuclear (^1^H-^13^C HSQC and HMBC) experiments were performed with quadrature phase detection in dimensions, using state-TPPI or QF detection mode in the indirect one. For each of the 512 increments in the indirect dimension, 2 K data points were collected and 32 or 64 transients were accumulated in the direct dimension. A ^13^C decoupling (GARP) was performed during acquisition time for heteronuclear experiments. A π/2 shifted square sine-bell function was applied in both dimensions before Fourier transformation. All NMR spectra were recorded at 300 K. Spectra were processed (phase and baseline correction) with Topspin version 3.5pl5.

Putative identifications for LC/MS and NMR discriminant variables were performed using web data base searches such as KEGG^33^ and HMDB^58^.

## Supplementary information

SUPPLEMENT

## Data Availability

NMR and MS data are available via 10.15454/Q7VKT5 and 10.15454/ZAWDKQ.
