# Supplementary material for: Metabolic modulations of Pseudomonas graminis in response to H2O2 in cloud water
Source: Sci Rep. 2019 Sep 5;9:12799. doi: 10.1038/s41598-019-49319-2 (PMC6728378; doi:10.1038/s41598-019-49319-2)
Supplement: Supplementary file 1 — SUPPLEMENT [file 41598_2019_49319_MOESM1_ESM.docx]

**SUPPLEMENT**

**Metabolic modulations of *Pseudomonas graminis* in response to H_2_O_2_ in cloud water**

Nolwenn Wirgot ^1^, Marie Lagrée ^1,2^, Mounir Traïkia ^1,2^, Ludovic Besaury^1^, Pierre Amato^1^, Isabelle Canet ^1^, Martine Sancelme ^1^, Cyril Jousse ^1,2^, Binta Diémé ^1,2^, Bernard Lyan ^2^ and Anne-Marie Delort ^1,2*^.

1 - Université Clermont Auvergne, CNRS, Sigma-Clermont, Institut de Chimie de Clermont-Ferrand F-63000 Clermont-Ferrand, France

2 - Plateforme d’Exploration du Métabolisme, Université Clermont Auvergne & I.N.R.A site de Theix, Clermont-Ferrand, France

*Corresponding author: A-marie.delort@uca.fr

**Table S1**: Mass spectrometry data of identified metabolites from LC-(ToF)-MS profiling. ID is an arbitrary identifier from XCMS software; M+X is the observed accurate mass; RT is the LC-retention time.

| **Identified metabolites** | **ID** | **RT (min)** | **Detected mass m/z** | **Theoretical mass m/z** | **Elemental formula** | **Ionic formula** |
| --- | --- | --- | --- | --- | --- | --- |
| **Amino acids** | | | | | | |
| **Isoleucine** | M132.1023T4 | 4.37 | 132.1023 | 132.1019 | C6H13NO2 | [M+H]+ |
| **Leucine** |  |  |  |  |  |  |
| **Valine** | M116.0719T3 | 2.73 | 116.0719 | 116.0706 | C5H11NO2 | [M-H]- |
| **Glutamate** | M146.0462T2 | 2.33 | 146.0462 | 146.0448 | C5H9NO4 | [M-H]- |
| **Methionine** | M150.0587T3 | 3.09 | 150.0587 | 150.0583 | C5H11NO2S | [M+H]+ |
| **Alanine** | M90.055T2 | 2.19 | 90.0550 | 90.0549 | C3H7NO2 | [M+H]+ |
| **Phenylalanine** | M167.0898T9 | 8.80 | 167.0898 | 167.0894 | C9H11NO2 | [M+H]+ |
| **Tyrosine** | M180.0662T3 | 3.28 | 180.0662 | 180.0655 | C9H11NO3 | [M-H]- |
| **Glycine** | M76.0388T2 | 2.15 | 76.0388 | 76.0393 | C2H5NO2 | [M+H]+ |
| **Saccharides** | | | | | | |
| **Trehalose/ Cellobiose** | M382.0827T2 | 2.37 | 382.0827 | 382.0828 | ^12^C11^13^CH22O11 | [M+K]+ |
| **Trehalose/Cellobiose 6-P** | M421.0756T2 | 2.38 | 421.0756 | 421.0742 | C12H23O14P | [M-H]- |
| **Xylose** | M185.0212T3 | 2.53 | 185.0212 | 185.0211 | C5H10O5 | [M+Cl]- |
| **Amines** | | | | | | |
| **Carnitine** | M162.1128T2 | 2.26 | 162.1128 | 162.1125 | C7H15NO3 | [M+H]+ |
| **Spermidine** | M146.1658T2 | 1.69 | 146.1658 | 146.1652 | C7H19N3 | [M+H]+ |
| **Lipids** | | | | | | |
| **Palmitoleic acid** | M255.23T20 | 19.81 | 255.23 | 255.2319 | C16H30O2 | [M+H]+ |
| **Lyso PE (16:1)** | M453.2808T15 | 15.35 | 453.2808 | 453.2805 | ^12^C20^13^CH42NO7P | [M+H]+ |
| **Lyso PC (16 :0)** | M496.3409 | 16.83 | 496.3402 | 496.3397 | C20H50NO7P | [M+H]+ |
| **Lyso PC (16:1)** | M494.3249T15 | 15.42 | 494.3249 | 494.3241 | C24H48NO7P | [M+H]+ |
| **Lyso PE (16:0)** | M455.2968T17 | 16.73 | 455.2968 | 455.2961 | ^12^C20^13^CH44NO7P | [M+H]+ |
| **Lyso PE (18:1)** | M481.3127T17 | 17.09 | 481.3127 | 481.3118 | ^12^C22^13^CH46NO7P | [M+H]+ |
| **Miscellaneous** | | | | | | |
| **Pantothenic acid** | M220.1173T9 | 8.97 | 220.1173 | 220.1179 | C9H17NO5 | [M+H]+ |
| **UMP** | M323.0291T3 | 3.19 | 323.0291 | 323.0275 | C9H13N2O9P | [M-H]- |
| **Glycerol 3-P** | M171.0064T2 | 2.35 | 171.0064 | 171.0053 | C3H9O6P | [M-H]- |
| **Betaine** | M118.0869T2 | 2.31 | 118.0869 | 118.0863 | C5H11NO2 | [M+H]+ |
| **Peptides** | | | | | | |
| **Alanyl-alanine** | M161.0929T2 | 2.40 | 161.0929 | 161.0921 | C6H12N2O3 | [M+H]+ |
| **Isoleucyl/leucyl-histidine** | M135.0851T3 | 2.54 | 135.0851 | 135.0840 | C12H20N4O3 | [M+2H]2+ |
| **Isoleucyl/leucyl-aspartic acid** | M247.128T3 | 3.12 | 247.1280 | 247.1288 | C10H18N2O5 | [M+H]+ |
| **Valyl-isoleucine/leucine** | M231.1694T10 | 10.38 | 231.1694 | 231.1703 | C11H22N2O3 | [M+H]+ |
| **Valyl-serine** | M205.1171T2 | 2.45 | 205.1171 | 205.1183 | C8H16N2O4 | [M+H]+ |
| **Valyl-aspartic acid** | M233.1126T3 | 2.50 | 233.1126 | 233.1132 | C9H16N2O5 | [M+H]+ |
| **Gln-Asp-Thr-Pro** | M460.2023T3 | 2.53 | 460.2023 | 460.2038 | C18H30N5O9 | [M+H]+ |
| **Arg-Cys-Ser-Trp** | M276.1247T17 | 16.73 | 276.1247 | 276.1234 | C23H34O6N8S | [M+2H]2+ |

**Table S2**: ^1^H and ^13^C chemical shifts (ppm) for all the metabolites detected, with the peak used for quantification highlighted. Chemical shifts for ^1^H in each metabolite are given with **bold** chemical shifts corresponding to the peaks labeled in the 1D ^1^H noesy and 2D ^1^H Jres spectra and with *italic* one when labeled in the 2D ^1^H-^1^H COSY and TOCSY. Chemical shifts for ^13^C are given with **bold** chemical shifts corresponding to the peaks labeled in the 2D ^1^H-^13^C HSQC spectrum. Other chemical shift estimations are given for H/C pairs that were not identified in the spectra due to overlap with other peaks or noise.

|  | Metabolite | ^1^H NMR | ^13^C NMR |
| --- | --- | --- | --- |
| 1 | Valine | **0.99** (d, 7 Hz), **1.05** (d, 7 Hz), **2.28** (m), **3.61** (d, 5 Hz) | 16.8, 17.9, 28.9, 61.3 |
| 2 | Leucine | **0.96** (d, 6 Hz), **0.97** (d, 6 Hz), *1.69*, *1.74* | **-** |
| 3 | Isoleucine | **0.94** (t, 7 Hz), **1.01** (d, 7 Hz), *1.27, 1.48*, *1.99* | 16.6 |
| 4 | Alanine | **1.48** (d, 7 Hz), **3.79** (q, 7 Hz) | - |
| 5 | Lysine | *1.45*, *1.69*, *1.89*, **3.01** (t, 7 Hz) | 39.4 |
| 6 | Glutamate | *2.04, 2.09, 2.33,* **3.76** (dd, 4 Hz, 7 Hz), | - |
| 7 | Tyrosine | *3.03*, *3.20*, *3.95*, **6.90** (d, 5 Hz), *7.20* | 117.1 |
| 8 | Glycine | **3.56** (s) | 41.4 |
| 9 | α-Glucose | **3.41** (dd, 10 Hz, 9 Hz), **3.53** (dd, 4 Hz, 10 Hz), *3.77*, *3.88*, **5.24** (d, 4 Hz) | 72.2, 91.9 |
| 10 | Trehalose | **3.46** (dd, 9 Hz, 10 Hz), **3.66** (dd, 4 Hz, 10 Hz), **3.77** (dd, 5 Hz, 12 Hz), **3.86** (t, 10 Hz), **3,87** (dd, 2 Hz, 12Hz), **5.20** (d, 4 Hz) | 60.8, 70.1, 71.4, 72.7, 93.4 |
| 11 | Glycogen | **3.32** (dd,9 Hz, 10 Hz), **3.58** (dd, 4 Hz, 10 Hz), **3.61** (dd, 4 Hz, 10 Hz), **3.77** (dd, 10 Hz, 8 Hz), *3.85*, **3.98** (dd, 9 Hz, 11 Hz), **5.42** (2d, 4 Hz) | 71.9, 73.2, 73.6, 99.8 |
| 12 | Putrescine | *1.77, 3.06* | 23.7, 39.0 |
| 14 | Glycerol | ***3.56*** (dd, 6 Hz, 12 Hz), ***3.68*** (dd, 4 Hz, 12 Hz) | 62.6, 75.7 |
| 15 | AMP | *4.81*, **6.15** (d, 6 Hz), **8.27** (s), **8.60** (s) | 74.6, 86.9 |
| 16 | ATP | *4.61*, **6.14** (d, 6 Hz), **8.27** (s), **8.59** (s) | 87.1 |
| 17 | Acetate | **1.92** (s) | 23.6 |
| 18 | Lactate | **1.33** (d, 7 Hz), **4.11** (q, 7 Hz) | 20.0 |
| 19 | Glutathione | *2.17*, *2.54*, 2.98 (dd, 10 Hz, 14 Hz) | 27.6, 31.5 |
| 20 | Methionine | **2.13** (s), *2.15*, *2.63*, **3.83** (dd, 5 Hz, 11 Hz) | 58.2 |
| 21 | β-Alanine | **2.56** (t, 7 Hz)**, 3.18** (t, 7 Hz) | **-** |
| 22 | Arginine | *1.68, 1.90, 3.76* | 54.6 |
| 23 | Proline | *2.08, 2.35* | 26.8, 33.5 |
| 24 | Aspartate | **2.68** (dd, 9 Hz, 17 Hz)**, 2.81** (dd, 4 Hz, 17 Hz)**, 3.91** (dd, 3 Hz, 8 Hz) | 36.4, 60.5 |
| 25 | Asparagine | **4.01** (dd, 4 Hz, 8 Hz)**, 2.87** (dd, 7 Hz, 17 Hz), *2.95* | - |
| 26 | Citrulline | *1.87***, 3.74** (dd, 5 Hz, 7 Hz) | - |
| 27 | Tryptophan | *3.45*, *4.02*, *3.27*, **7.54** (d, 8 Hz), **7.72** (d, 8 Hz) | - |
| 28 | Cellobiose | **3.75** (dd, 5 Hz, 12 Hz), **4.52** (d, 8 Hz), **5.24** (d, 4 Hz) | 65.3, 92.1, 102.6 |
| 29 | Fructose | **3.88** (d, 3 Hz), **3.73** (m) | 71.1 |
| 30 | Maltose | **5.40** (d, 4 Hz), **5.23** (d, 4 Hz), **3.41** (t, 10 Hz), | 72.8, 92.1, 99.8 |
| 31 | Carnitine | *2.42*, **3.21** (s) | 54.0 |
| 32 | Succinate | **2.40** (s) | 34.2 |
| 33 | Citrate | *2.52 (dd), 2.66 (dd)* | **-** |
| 34 | β-hydroxybutyrate | **1.20** (d, 6 Hz), *2.32*, *2.42*, *4.15,* | **-** |
| 35 | Pipecolic acid | *1.64, 2.24, 2.94* | 43.5 |
| 36 | Pantothenic acid | *2.42***, 3.39** (d, 11 Hz), *3.44*, *3.52* | 73.7 |
| 37 | Inosinic acid | *4.03***,** *4.38***, 4.52** (m)**, 8.23** (s), **8.55** (s), **6.14** (d, 6 Hz) | 63.9, 85.3 |
| 38 | Nicotinamide ribotide | *4.49, 4.61, 6.19, 8.32, 9.01* | **-** |
| 39 | NADH | **4.38** (s), **8.20** (s), **8.46** (s) | **-** |
| 40 | UMP | **4.36** (t, 5 Hz), **4.42** (t, 5 Hz), *5.99***, 8.09** (d, 8 Hz) | 73.9 |
| 41 | Betaine | **3.27** (s), **3.91** (s) | 53.5, 68.2 |

**Table S3**: Identification criteria used for metabolite identifications considering MS and NMR experiments according to know references (databases or commercial standards when mentioned). Matches between the signals observed and references are darkened. MS experiments: TOF, accurate mass measurement on LC-ToF (Time of Flight); STD, comparison using pure chemical standard (similar parent ion masses and retention times). NMR experiments (validation mark for at least one result in the following experiment): ^1^H, chemical shift on proton NMR experiment; Jres, chemical shift and coupling constant on J-resolved experiment; COSY, chemical shift and correlation on COrrelationSpectrocopY experiment; TOCSY, chemical shift and correlation on TOtal Correlation SpectroscopY experiment; HSQC, chemical shift and correlation on Heteronuclear Single Quantum Correlation experiment.

|  | **MS** | |  |  | **NMR** | | | | |
| --- | --- | --- | --- | --- | --- | --- | --- | --- | --- |
| **Identified VIP Metabolite** | **TOF** | **STD** | **HRMS** | **MS/MS** | **^1^H** | **Jres** | **COSY** | **TOCSY** | **HSQC** |
| Isoleucine* |  |  |  |  |  |  |  |  |  |
| Leucine* |  |  |  |  |  |  |  |  |  |
| Valine |  |  |  |  |  |  |  |  |  |
| Lysine |  |  |  |  |  |  |  |  |  |
| Arginine |  |  |  |  |  |  |  |  |  |
| Proline |  |  |  |  |  |  |  |  |  |
| Glutamate |  |  |  |  |  |  |  |  |  |
| Methionine |  |  |  |  |  |  |  |  |  |
| Alanine |  |  |  |  |  |  |  |  |  |
| β-Alanine |  |  |  |  |  |  |  |  |  |
| Aspartate |  |  |  |  |  |  |  |  |  |
| Asparagine |  |  |  |  |  |  |  |  |  |
| Phenylalanine |  |  |  |  |  |  |  |  |  |
| Tyrosine |  |  |  |  |  |  |  |  |  |
| Glycine |  |  |  |  |  |  |  |  |  |
| Citrulline |  |  |  |  |  |  |  |  |  |
| Tryptophan |  |  |  |  |  |  |  |  |  |
| Trehalose* |  |  |  |  |  |  |  |  |  |
| Trehalose 6-P* |  |  |  |  |  |  |  |  |  |
| Cellobiose* |  |  |  |  |  |  |  |  |  |
| Glycogen |  |  |  |  |  |  |  |  |  |
| α-glucose |  |  |  |  |  |  |  |  |  |
| Fructose |  |  |  |  |  |  |  |  |  |
| Maltose |  |  |  |  |  |  |  |  |  |
| Xylose |  |  |  |  |  |  |  |  |  |
| Putrescine |  |  |  |  |  |  |  |  |  |
| Carnitine |  |  |  |  |  |  |  |  |  |
| Spermidine |  |  |  |  |  |  |  |  |  |
| Palmitoleic acid |  |  |  |  |  |  |  |  |  |
| Lyso PC (16 :0) |  |  |  |  |  |  |  |  |  |
| Lyso PE (16:1) |  |  |  |  |  |  |  |  |  |
| Lyso PC (16:1) |  |  |  |  |  |  |  |  |  |
| Lyso PE (16:0) |  |  |  |  |  |  |  |  |  |
| Lyso PE (18:1) |  |  |  |  |  |  |  |  |  |
| Acetate |  |  |  |  |  |  |  |  |  |
| Lactate |  |  |  |  |  |  |  |  |  |
| Succinate |  |  |  |  |  |  |  |  |  |
| Citrate |  |  |  |  |  |  |  |  |  |
| β-hydroxybutyrate |  |  |  |  |  |  |  |  |  |
| Pipecolic acid |  |  |  |  |  |  |  |  |  |
| Pantothenic acid |  |  |  |  |  |  |  |  |  |
| Inosinic acid |  |  |  |  |  |  |  |  |  |
| Glutathione (GSH) |  |  |  |  |  |  |  |  |  |
| Nicotinamide ribotide |  |  |  |  |  |  |  |  |  |
| AMP |  |  |  |  |  |  |  |  |  |
| ATP |  |  |  |  |  |  |  |  |  |
| NADH |  |  |  |  |  |  |  |  |  |
| UMP |  |  |  |  |  |  |  |  |  |
| Glycerol |  |  |  |  |  |  |  |  |  |
| Glycerol 3-P |  |  |  |  |  |  |  |  |  |
| Betaine |  |  |  |  |  |  |  |  |  |
| Alanyl-alanine |  |  |  |  |  |  |  |  |  |
| Isoleucyl/leucyl-histidine |  |  |  |  |  |  |  |  |  |
| Isoleucyl/leucyl-aspartic acid |  |  |  |  |  |  |  |  |  |
| Valyl-isoleucine/leucine |  |  |  |  |  |  |  |  |  |
| Valyl-serine |  |  |  |  |  |  |  |  |  |
| Valyl-aspartic acid |  |  |  |  |  |  |  |  |  |
| Gln-Asp-Thr-Pro |  |  |  |  |  |  |  |  |  |
| Arg-Cys-Ser-Trp |  |  |  |  |  |  |  |  |  |

**Table S4:** Composition of marine artificial cloud solution. Stocks solutions were prepared in ultrapure water (resistivity of 18.2 MΩ.cm^-1^).

| **Compound** | **Elemental formula** | **Concentration (µM)** | **Supplier** |
| --- | --- | --- | --- |
| Acetic acid | C_2_H_4_O_2_ | 20 | Acros organics |
| Formic acid | CH_2_O_2_ | 15 | Fluka Analytical |
| Oxalic acid | C_2_H_2_O_4_ | 3 | Fluka Analytical |
| Succinic acid | C_4_H_6_O_4_ | 1.5 | Fluka Analytical |
| Ammonium nitrate | NH_4_NO_3_ | 80 | Fluka Analytical |
| Magnesium chloride hexahydrate | MgCl_2_, 6H_2_O | 10 | Sigma-Aldrich |
| Potassium sulfate | K_2_SO_4_ | 5 | Fluka Analytical |
| Calcium chloride dihydrate | CaCl_2_, 2H_2_O | 40 | Sigma-Aldrich |
| Sodium chloride | NaCl | 200 | Sigma-Aldrich |

**Table S5:** Matrix of relative intensities for all samples (Stressed and Reference) corresponding to each identified metabolite obtained for LC-MS analyses. Means, standard deviations and ratios S/R were calculated.

| **Identified compounds** | Glycine | Alanine | Betaine | Isoleucine/leucine | Isoleucyl/leucyl-histidine | Spermidine | Methionine | Ala-ala | Carnitine | Phenylalanine |
| --- | --- | --- | --- | --- | --- | --- | --- | --- | --- | --- |
| **Samples / Ions** | M76.0388T2 | M90.055T2 | M118.0869T2 | M132.1023T4 | M135.0851T2 | M146.1658T1 | M150.0587T3 | M161.0929T2 | M162.1128T2 | M167.0898T8 |
| **Stressed (S) (50 min)** | 2862.790991 | 2290.932137 | 319614.8321 | 7344.860642 | 278.8990116 | 1529.343871 | 22243.63217 | 549.8581508 | 1412.683967 | 2670.925439 |
|  | 1581.934375 | 756.7838781 | 410050.9159 | 4865.410517 | 630.1194089 | 4188.156773 | 34586.8453 | 540.6776279 | 1924.622647 | 1842.84066 |
|  | 2205.508735 | 732.6635855 | 290532.0321 | 12138.89478 | 1007.458352 | 1664.085278 | 29345.3323 | 401.8509974 | 4064.688469 | 1959.9976 |
|  | 2879.9402 | 3058.079479 | 253044.4529 | 9200.134616 | 599.3979854 | 2856.515547 | 23546.61112 | 537.4213938 | 2291.84998 | 2443.094227 |
|  | 1970.442469 | 1813.581112 | 332640.0923 | 6213.685386 | 556.1105845 | 4813.254409 | 33633.64047 | 512.5900082 | 2357.242224 | 1828.390131 |
|  | 3460.025191 | 2233.48744 | 352123.9807 | 8593.329861 | 1097.42295 | 3093.557138 | 24739.49851 | 513.2394203 | 5436.968645 | 1909.589903 |
|  | 3459.011922 | 3279.440764 | 278395.8651 | 10314.54913 | 648.0178996 | 2687.847637 | 22140.25726 | 537.8209367 | 1846.753074 | 2101.079923 |
|  | 2574.352899 | 3122.493308 | 406621.8553 | 7173.177187 | 716.128975 | 5298.585264 | 32750.98485 | 590.9547873 | 2984.91226 | 1987.05361 |
|  | 2474.344335 | 2411.109498 | 282661.9164 | 10901.02895 | 1172.378901 | 3161.387968 | 30583.92048 | 443.6621205 | 4681.507276 | 2067.826981 |
| **Reference (R) (50 min)** | 1604.039037 | 2385.310171 | 28900.58871 | 2990.24783 | 270.5114587 | 2196.428911 | 15032.96996 | 721.6687728 | 561.6554377 | 1003.627327 |
|  | 1558.963255 | 2663.6155 | 71572.21674 | 3895.100436 | 317.0904213 | 2677.045721 | 21745.09186 | 789.1134791 | 2131.318996 | 933.2516709 |
|  | 1879.348288 | 3787.47937 | 77497.16855 | 4258.012828 | 219.6883188 | 1354.618302 | 19876.45871 | 653.3392743 | 3037.181244 | 719.7136544 |
|  | 2131.925948 | 3790.960321 | 51819.3848 | 3327.044002 | 430.3926088 | 2430.118987 | 18574.5033 | 996.5894461 | 1018.385047 | 1166.538796 |
|  | 2137.600795 | 3748.361166 | 68510.35644 | 3255.702717 | 301.3584283 | 3119.869016 | 23281.92938 | 994.2414994 | 1769.551163 | 1202.962164 |
|  | 1851.679533 | 2020.311994 | 86714.24983 | 3276.531007 | 325.5386696 | 2906.991902 | 19951.35825 | 742.6068992 | 2098.404644 | 663.6968198 |
|  | 2062.779614 | 2964.922084 | 28400.58297 | 4086.570814 | 611.3016082 | 157.27442 | 17420.69682 | 908.0970746 | 1112.07694 | 1106.478089 |
|  | 1827.090807 | 3076.715617 | 66478.24346 | 3080.377332 | 181.1253346 | 3920.473948 | 25079.74821 | 766.4874237 | 1409.71828 | 685.0136203 |
|  | 1902.515514 | 2861.296095 | 89839.34613 | 2934.02315 | 201.3089856 | 1294.381246 | 18637.38062 | 505.4293711 | 2187.540757 | 719.4392995 |
| **"R" mean** | 1.88E+03 | 3.03E+03 | 6.33E+04 | 3.46E+03 | 3.18E+02 | 2.23E+03 | 2.00E+04 | 7.86E+02 | 1.70E+03 | 9.11E+02 |
| **"S" mean** | 2.61E+03 | 2.19E+03 | 3.25E+05 | 8.53E+03 | 7.45E+02 | 3.25E+03 | 2.82E+04 | 5.14E+02 | 3.00E+03 | 2.09E+03 |
| **"R" Standard deviation** | 2.08E+02 | 6.40E+02 | 2.26E+04 | 4.94E+02 | 1.34E+02 | 1.13E+03 | 3.06E+03 | 1.60E+02 | 7.55E+02 | 2.19E+02 |
| **"S" Standard deviation** | 6.35E+02 | 9.48E+02 | 5.60E+04 | 2.35E+03 | 2.90E+02 | 1.30E+03 | 5.05E+03 | 5.76E+01 | 1.41E+03 | 2.86E+02 |
| **Ratio S/R** | 1.38 | 0.72 | 5.14 | 2.47 | 2.35 | 1.46 | 1.41 | 0.65 | 1.76 | 2.29 |

| **Identified compounds** | Valine-Serine | Acide pantothenique | Valine-Isoleucine/leucine | Valine-Acide aspartique | Isoleucyl/leucyl-aspartic acid | Acide palmitoleique | Arg-Cys-Ser-Trp | Trehalose/Cellobiose | Lyso PE (16:1) | Lyso PE (16:0) |
| --- | --- | --- | --- | --- | --- | --- | --- | --- | --- | --- |
| **Samples / Ions** | M205.1171T2 | M220.1173T8 | M231.1694T10 | M233.1126T2 | M247.128T3 | M255.23T19 | M276.1247T16 | M382.0827T2 | M453.2808T15 | M455.2968T16 |
| **Stressed (S) (50 min)** | 2280.35843 | 1918.112034 | 358.5406836 | 1261.33625 | 845.212685 | 341.519241 | 295.4552282 | 811.6807254 | 2221.632602 | 2982.021323 |
|  | 3116.940215 | 2758.863334 | 1050.289436 | 1900.390313 | 1855.295833 | 245.1331062 | 287.3800853 | 908.0941746 | 2235.296401 | 3339.740237 |
|  | 3309.164184 | 2390.383939 | 3281.1474 | 2228.139959 | 2356.718044 | 0 | 166.2987633 | 887.6735569 | 1616.338825 | 1663.112208 |
|  | 1554.704381 | 1937.611695 | 414.6495214 | 1911.783924 | 1755.498336 | 434.4102061 | 526.5430385 | 875.1824691 | 2866.971997 | 5196.534162 |
|  | 2231.10397 | 2939.991458 | 1171.633968 | 2342.937166 | 2423.614552 | 308.8724963 | 507.7340568 | 991.2457497 | 2105.181167 | 3134.797272 |
|  | 5322.478832 | 2045.733622 | 2481.298623 | 2506.175395 | 1529.909764 | 0 | 0 | 765.3612059 | 1176.515332 | 1293.807547 |
|  | 1743.084893 | 1968.261304 | 499.5190199 | 2034.001655 | 1560.155448 | 270.1389191 | 309.9702749 | 658.908924 | 2195.00273 | 4399.642254 |
|  | 2265.886258 | 3140.408773 | 1285.98398 | 2960.89938 | 2287.90994 | 269.121534 | 353.8194224 | 860.0312228 | 2693.443431 | 5096.468307 |
|  | 3408.910126 | 2417.990406 | 3141.417269 | 1928.005315 | 1811.506476 | 0 | 390.4504589 | 921.5405003 | 1882.693754 | 3197.101047 |
| **Reference (R) (50 min)** | 2051.979229 | 702.4121701 | 0 | 827.716098 | 260.5865764 | 724.5355592 | 530.3715881 | 713.7302247 | 3858.890401 | 8704.812969 |
|  | 4233.143559 | 1337.81255 | 563.2239296 | 2189.593595 | 389.7564544 | 320.3653988 | 358.3956508 | 1319.388313 | 3128.757977 | 4858.913885 |
|  | 3187.423222 | 1540.360331 | 385.3121906 | 1146.735102 | 276.6911547 | 183.0693817 | 306.4439596 | 1208.01816 | 3264.507981 | 4232.70656 |
|  | 4572.155683 | 780.3684783 | 0 | 970.2029805 | 184.1788453 | 617.2374059 | 523.9342423 | 1267.83682 | 4953.380388 | 7509.72507 |
|  | 5235.04216 | 1669.037724 | 586.0245008 | 1135.94677 | 205.6785917 | 577.5452992 | 669.7730867 | 1229.02025 | 5120.475922 | 9541.090362 |
|  | 4369.979696 | 1577.5197 | 329.4744234 | 1143.90634 | 441.7356677 | 556.9651614 | 625.2398703 | 1322.644697 | 5432.059268 | 9077.750475 |
|  | 2937.295104 | 824.0353582 | 253.8639274 | 855.0560883 | 309.9513933 | 361.4345016 | 427.879486 | 1014.887139 | 2806.438491 | 4272.990981 |
|  | 4404.018408 | 1377.642522 | 352.4059139 | 868.9181777 | 358.0916644 | 1102.909752 | 833.3966702 | 1159.509946 | 8448.205382 | 14729.22236 |
|  | 4793.763466 | 1535.529951 | 329.115765 | 656.8426955 | 250.5190885 | 696.2147721 | 691.0835617 | 1182.847238 | 6242.630085 | 10624.09 |
| **"R" mean** | 3.98E+03 | 1.26E+03 | 3.11E+02 | 1.09E+03 | 2.97E+02 | 5.71E+02 | 5.52E+02 | 1.16E+03 | 4.81E+03 | 8.17E+03 |
| **"S" mean** | 2.80E+03 | 2.39E+03 | 1.52E+03 | 2.12E+03 | 1.83E+03 | 2.08E+02 | 3.15E+02 | 8.53E+02 | 2.11E+03 | 3.37E+03 |
| **"R" Standard deviation** | 1.03E+03 | 3.83E+02 | 2.07E+02 | 4.46E+02 | 8.55E+01 | 2.70E+02 | 1.70E+02 | 1.91E+02 | 1.80E+03 | 3.43E+03 |
| **"S" Standard deviation** | 1.15E+03 | 4.65E+02 | 1.15E+03 | 4.73E+02 | 4.97E+02 | 1.65E+02 | 1.63E+02 | 9.72E+01 | 5.14E+02 | 1.36E+03 |
| **Ratio S/R** | 0.71 | 1.90 | 4.89 | 1.95 | 6.14 | 0.36 | 0.57 | 0.74 | 0.44 | 0.41 |

| **Identified compounds** | Gln-Asp-Thr-Pro | Lyso PE (18:1) | Lyso PC (16:1) | Valine | Glycerol 3-P | Tyrosine | Xylose | UMP | Trehalose/Cellobiose 6-P | Lyso PC (16 :0) |
| --- | --- | --- | --- | --- | --- | --- | --- | --- | --- | --- |
| **Samples / Ions** | M460.2023T2 | M481.3127T17 | M494.3249T15 | M116.0719T2 | M171.0064T2 | M180.0662T3 | M185.0212T2 | M323.0291T3 | M421.0756T2 | M455.2968T16,73 |
| **Stressed (S) (50 min)** | 3534.484122 | 2874.591769 | 1941.632958 | 2382.902513 | 1544.93687 | 1497.736289 | 314.9043174 | 1418.684374 | 4302.459837 | 2982.021323 |
|  | 5183.553701 | 2508.489974 | 1367.02343 | 2752.797407 | 1029.900857 | 2585.271195 | 307.0450328 | 1499.573087 | 5557.254535 | 3339.740237 |
|  | 8884.375334 | 1648.740784 | 1230.062386 | 2754.097285 | 870.8837208 | 1938.562523 | 207.824432 | 1292.208504 | 5032.004139 | 1663.112208 |
|  | 5432.744674 | 4838.392082 | 2207.576334 | 2273.982763 | 2014.95985 | 2138.512956 | 512.8326146 | 1390.42744 | 4126.043503 | 5196.534162 |
|  | 8239.837236 | 2738.914809 | 1270.177773 | 2708.689273 | 971.1611475 | 3303.258767 | 372.1845616 | 1909.646196 | 6678.688015 | 3134.797272 |
|  | 4929.165675 | 1002.174194 | 1495.801812 | 2564.396313 | 785.8016946 | 1374.744552 | 0 | 1204.284797 | 6295.560663 | 1293.807547 |
|  | 4183.840036 | 2658.045944 | 2284.953996 | 2409.402504 | 1594.955064 | 2202.6215 | 317.5776019 | 1505.559396 | 4536.701321 | 4399.642254 |
|  | 8284.721509 | 3396.962416 | 1630.306225 | 3980.604288 | 1521.300111 | 3509.597679 | 502.2019801 | 2181.697559 | 6706.357629 | 5096.468307 |
|  | 6914.350137 | 2449.753468 | 2469.985583 | 3563.412315 | 903.6304502 | 1782.477796 | 204.682345 | 1486.742295 | 5039.304438 | 3197.101047 |
| **Reference (R) (50 min)** | 528.7662088 | 7036.217076 | 2425.77391 | 365.4541315 | 3033.972605 | 1016.082453 | 724.6516386 | 2498.399644 | 7994.434232 | 8704.812969 |
|  | 1468.193045 | 4047.883943 | 2019.818623 | 602.1266218 | 1690.185311 | 1766.706859 | 555.2318076 | 4340.856853 | 11324.3167 | 4858.913885 |
|  | 2635.086401 | 3474.594395 | 1876.045238 | 739.4144474 | 1512.987768 | 1034.990687 | 345.1422202 | 3180.845416 | 13289.41513 | 4232.70656 |
|  | 638.9211464 | 6493.361544 | 3376.515036 | 416.8221038 | 3675.420223 | 0 | 541.4540283 | 3579.095896 | 10303.77561 | 7509.72507 |
|  | 908.3133758 | 7756.117936 | 2723.58315 | 470.2701246 | 2861.179354 | 1274.876115 | 839.5368828 | 3924.277078 | 11374.37118 | 9541.090362 |
|  | 2345.286199 | 7612.073975 | 3831.027292 | 679.0400377 | 1954.453944 | 890.4637112 | 605.4436007 | 2822.923671 | 10878.36318 | 9077.750475 |
|  | 982.6966628 | 3776.33259 | 1688.925733 | 398.1720432 | 2195.870575 | 1199.694669 | 589.9192743 | 3524.66857 | 9258.832471 | 4272.990981 |
|  | 1122.861939 | 11641.661 | 3402.103319 | 538.1561357 | 3402.187936 | 1555.733987 | 1225.781114 | 3248.483194 | 8841.699609 | 14729.22236 |
|  | 2256.097334 | 8946.637896 | 2972.746384 | 880.5149003 | 2449.457329 | 949.6623166 | 650.9862491 | 2726.178713 | 9746.457756 | 10624.09 |
| **"R" mean** | 1.43E+03 | 6.75E+03 | 2.70E+03 | 5.66E+02 | 2.53E+03 | 1.08E+03 | 6.75E+02 | 3.32E+03 | 1.03E+04 | 8.17E+03 |
| **"S" mean** | 6.18E+03 | 2.68E+03 | 1.77E+03 | 2.82E+03 | 1.25E+03 | 2.26E+03 | 3.04E+02 | 1.54E+03 | 5.36E+03 | 3.37E+03 |
| **"R" Standard deviation** | 7.89E+02 | 2.68E+03 | 7.54E+02 | 1.75E+02 | 7.60E+02 | 4.97E+02 | 2.47E+02 | 5.93E+02 | 1.59E+03 | 3.43E+03 |
| **"S" Standard deviation** | 1.96E+03 | 1.07E+03 | 4.71E+02 | 5.75E+02 | 4.29E+02 | 7.47E+02 | 1.58E+02 | 3.09E+02 | 1.00E+03 | 1.36E+03 |
| **Ratio S/R** | 4.31 | 0.40 | 0.65 | 4.99 | 0.49 | 2.10 | 0.45 | 0.47 | 0.52 | 0.41 |

**Table S7:** Matrix of relative intensities for all samples (Stressed and Reference) corresponding to each identified metabolite obtained for NMR analyses. Means, standard deviations and ratios S/R were calculated.

| **Identified compounds** | Nicotinamide ribotide | ATP | UMP | Glycogen | Maltose | Cellobiose | Inosinic acid | NADH | Fructose | Lactate |  |
| --- | --- | --- | --- | --- | --- | --- | --- | --- | --- | --- | --- |
| **Samples / Buckets** | 6.195 | 6.135 | 5.985 | 5.425 | 5.395 | 4.655 | 4.505 | 4.355 | 4.125 | 4.115 |  |
| **Stressed (S) (50 min)** | 0.00010216 | 0.00009439 | 0.00014164 | 0.00044605 | 0.00198155 | 0.00004962 | 0.00026968 | 0.00033932 | 0.00033422 | 0.00034699 |  |
|  | 0.00010789 | 0.00009109 | 0.00023103 | 0.00067593 | 0.00197754 | 0.00004289 | 0.00050525 | 0.00042187 | 0.00015772 | 0.00019666 |  |
|  | 0.00006826 | 0.00014982 | 0.00026238 | 0.00080369 | 0.00252371 | 0.00003949 | 0.00080441 | 0.00081847 | 0.00070408 | 0.00072467 |  |
|  | 0.00007175 | 0.00009487 | 0.00017486 | 0.0005211 | 0.00222662 | 0.0000436 | 0.0002816 | 0.00036747 | 0.00036496 | 0.00043279 |  |
|  | 0.00006587 | 0.00011373 | 0.00027999 | 0.00077082 | 0.00221443 | 0.00004709 | 0.00061077 | 0.00059394 | 0.00040575 | 0.00045095 |  |
|  | 0.00024112 | 0.00009577 | 0.00006079 | 0.00046076 | 0.00242562 | 0.00018322 | 0.00038893 | 0.0003708 | 0.00014781 | 0.00011139 |  |
|  | 0.00006988 | 0.00010829 | 0.00015315 | 0.00046343 | 0.00210637 | 0.0000449 | 0.00026324 | 0.00035547 | 0.00032781 | 0.00037351 |  |
|  | 0.00010217 | 0.00009885 | 0.00023165 | 0.0007102 | 0.00221629 | 0.00002583 | 0.00058988 | 0.00048577 | 0.00035109 | 0.00038297 |  |
|  | 0.00010966 | 0.00012316 | 0.00025443 | 0.00066853 | 0.00253079 | 0.00003791 | 0.00054549 | 0.00051946 | 0.0003609 | 0.00040609 |  |
| **Reference (R) (50 min)** | 0.00018577 | 0.00023715 | 0.00046267 | 0.00072482 | 0.00073304 | 0.00005725 | 0.00048695 | 0.00067119 | 0.00033392 | 0.0004124 |  |
|  | 0.00012845 | 0.00025788 | 0.00060018 | 0.0011057 | 0.00102221 | 0.00004156 | 0.00088808 | 0.00084097 | 0.00027783 | 0.0003197 |  |
|  | 0.00003771 | 0.00035932 | 0.00048073 | 0.00134318 | 0.00247474 | 0.00003047 | 0.00077136 | 0.00071929 | 0.00031367 | 0.00036525 |  |
|  | 0.00014719 | 0.00016211 | 0.00044419 | 0.00072772 | 0.00079899 | 0.00004144 | 0.00045292 | 0.00055259 | 0.00013512 | 0.00019557 |  |
|  | 0.00010231 | 0.00022526 | 0.00048188 | 0.00095869 | 0.00092737 | 0.00004385 | 0.0006568 | 0.00057227 | 0.00006003 | 0.00008703 |  |
|  | 0.00011836 | 0.00026158 | 0.00037048 | 0.00099667 | 0.00234384 | 0.00004369 | 0.00071181 | 0.00055263 | 0.0001774 | 0.00022626 |  |
|  | 0.00009395 | 0.00025624 | 0.00051978 | 0.00081463 | 0.00093662 | 0.00005617 | 0.0007617 | 0.00080148 | 0.00046653 | 0.00051979 |  |
|  | 0.00009994 | 0.00028385 | 0.00048399 | 0.00086821 | 0.00085995 | 0.00007073 | 0.00062969 | 0.00050263 | 0.00006741 | 0.0001001 |  |
|  | 0.00017386 | 0.0002283 | 0.0003211 | 0.00093307 | 0.00213278 | 0.00003956 | 0.00063977 | 0.00049538 | 0.00015342 | 0.00019613 |  |
| **"R" mean** | 1.21E-04 | 2.52E-04 | 4.63E-04 | 9.41E-04 | 1.36E-03 | 4.59E-05 | 6.67E-04 | 6.34E-04 | 2.21E-04 | 2.69E-04 |  |
| **"S" mean** | 1.04E-04 | 1.08E-04 | 1.99E-04 | 6.13E-04 | 2.24E-03 | 5.80E-05 | 4.73E-04 | 4.75E-04 | 3.50E-04 | 3.81E-04 |  |
| **"R" Standard deviation** | 4.50E-05 | 5.27E-05 | 8.08E-05 | 1.96E-04 | 7.29E-04 | 1.22E-05 | 1.37E-04 | 1.29E-04 | 1.36E-04 | 1.46E-04 |  |
| **"S" Standard deviation** | 5.45E-05 | 1.90E-05 | 7.14E-05 | 1.41E-04 | 2.10E-04 | 4.77E-05 | 1.86E-04 | 1.55E-04 | 1.61E-04 | 1.71E-04 |  |
| **Ratio S/R** | 0.86 | 0.43 | 0.43 | 0.65 | 1.65 | 1.26 | 0.71 | 0.75 | 1.59 | 1.41 |  |
|  |  |  |  |  |  |  |  |  |  |  | |
| **Identified compounds** | AMP | Asparagine | Tyrosine | Betaine | Alanine | Glycerol | α-glucose | Glycine | Trehalose | Tryptophan |  |
| **Samples / Buckets** | 4.015 | 4.005 | 3.955 | 3.895 | 3.775 | 3.695 | 3.595 | 3.555 | 3.455 | 3.265 |  |
| **Stressed (S) (50 min)** | 0.00100046 | 0.00087482 | 0.00185704 | 0.00897787 | 0.0243473 | 0.00198317 | 0.00406848 | 0.00187845 | 0.00400621 | 0.01405605 |  |
|  | 0.00110678 | 0.00096552 | 0.00181452 | 0.01349315 | 0.02342266 | 0.00186959 | 0.00556048 | 0.00224603 | 0.00443752 | 0.02149473 |  |
|  | 0.00180415 | 0.00152599 | 0.00289345 | 0.01257882 | 0.02447634 | 0.00182958 | 0.00452719 | 0.00163515 | 0.00422657 | 0.01942951 |  |
|  | 0.00099296 | 0.00082055 | 0.00189011 | 0.00945479 | 0.02353653 | 0.00202754 | 0.00432091 | 0.0019132 | 0.00393013 | 0.01303719 |  |
|  | 0.00141845 | 0.00122889 | 0.00221666 | 0.01401588 | 0.0227186 | 0.00193674 | 0.00551446 | 0.00223029 | 0.00447683 | 0.01859396 |  |
|  | 0.00108929 | 0.0008127 | 0.00207032 | 0.01220573 | 0.02331517 | 0.00159123 | 0.00485221 | 0.0015548 | 0.00368178 | 0.01899261 |  |
|  | 0.00092639 | 0.00076227 | 0.00176036 | 0.0095643 | 0.02380223 | 0.00199507 | 0.00427363 | 0.00179068 | 0.00372208 | 0.01477935 |  |
|  | 0.00134732 | 0.00119362 | 0.00212908 | 0.01417905 | 0.02261271 | 0.00198893 | 0.00560021 | 0.00231247 | 0.00462917 | 0.01976277 |  |
|  | 0.00122109 | 0.0009372 | 0.00228385 | 0.01192291 | 0.02373177 | 0.00187435 | 0.0051471 | 0.00189806 | 0.00389443 | 0.01964822 |  |
| **Reference (R) (50 min)** | 0.00171369 | 0.00132482 | 0.00133262 | 0.00232089 | 0.02526427 | 0.00172884 | 0.00192226 | 0.00151861 | 0.00401353 | 0.00199183 |  |
|  | 0.00205379 | 0.00150581 | 0.00144814 | 0.00304296 | 0.02590986 | 0.00200238 | 0.00319156 | 0.00219608 | 0.00473572 | 0.00299182 |  |
|  | 0.00207288 | 0.00149408 | 0.00221982 | 0.00350919 | 0.03087998 | 0.00234119 | 0.00374382 | 0.00210563 | 0.00523829 | 0.00283925 |  |
|  | 0.00147288 | 0.00108002 | 0.00101862 | 0.00162382 | 0.02482585 | 0.00204959 | 0.00258188 | 0.00187962 | 0.00470353 | 0.0020784 |  |
|  | 0.00140331 | 0.00095535 | 0.00084509 | 0.00205303 | 0.02482099 | 0.00223555 | 0.00366599 | 0.00261759 | 0.00507508 | 0.00302574 |  |
|  | 0.00163119 | 0.00117781 | 0.00195941 | 0.00313258 | 0.02864212 | 0.00235662 | 0.00384761 | 0.00223704 | 0.00502074 | 0.0025869 |  |
|  | 0.00199482 | 0.00164799 | 0.00161137 | 0.00280959 | 0.02705324 | 0.00175195 | 0.00195589 | 0.00143013 | 0.00476151 | 0.00187452 |  |
|  | 0.00145812 | 0.00089912 | 0.00090452 | 0.00239904 | 0.02565389 | 0.00226234 | 0.00397708 | 0.00284357 | 0.00500868 | 0.00287967 |  |
|  | 0.001437 | 0.00103945 | 0.00176303 | 0.00296393 | 0.0282229 | 0.00228674 | 0.00392872 | 0.00236885 | 0.00508197 | 0.00262273 |  |
| **"R" mean** | 1.69E-03 | 1.24E-03 | 1.46E-03 | 2.65E-03 | 2.68E-02 | 2.11E-03 | 3.20E-03 | 2.13E-03 | 4.85E-03 | 2.54E-03 |  |
| **"S" mean** | 1.21E-03 | 1.01E-03 | 2.10E-03 | 1.18E-02 | 2.36E-02 | 1.90E-03 | 4.87E-03 | 1.94E-03 | 4.11E-03 | 1.78E-02 |  |
| **"R" Standard deviation** | 2.79E-04 | 2.68E-04 | 4.79E-04 | 5.95E-04 | 2.07E-03 | 2.43E-04 | 8.39E-04 | 4.67E-04 | 3.63E-04 | 4.49E-04 |  |
| **"S" Standard deviation** | 2.77E-04 | 2.52E-04 | 3.50E-04 | 2.02E-03 | 6.36E-04 | 1.34E-04 | 6.04E-04 | 2.71E-04 | 3.44E-04 | 2.99E-03 |  |
| **Ratio S/R** | 0.72 | 0.82 | 1.44 | 4.46 | 0.88 | 0.90 | 1.52 | 0.91 | 0.85 | 6.98 |  |
|  |  |  |  |  |  |  |  |  |  |  |  |
| **Identified compounds** | Putrescine | Glutathione | Aspartate | Citrate | Methionine | β-Alanine | Carnitine | Succinate/  Pantothenic acid | β-hydroxybutyrate | Pipecolic acid |  |
| **Samples / Buckets** | 3.015 | 2.985 | 2.675 | 2.655 | 2.645 | 2.555 | 2.415 | 2.405 | 2.295 | 2.235 |  |
| **Stressed (S) (50 min)** | 0.00034458 | 0.00024537 | 0.00048447 | 0.00054321 | 0.00031088 | 0.00069158 | 0.00004437 | 0.00048038 | 0.00090407 | 0.00072862 |  |
|  | 0.00061754 | 0.00025226 | 0.00035536 | 0.00053754 | 0.00031213 | 0.00071123 | 0.0000385 | 0.00033316 | 0.00097406 | 0.00078634 |  |
|  | 0.00042856 | 0.00015942 | 0.00040822 | 0.00065765 | 0.00037439 | 0.00128912 | 0.00003645 | 0.0006758 | 0.00099176 | 0.00068374 |  |
|  | 0.00043445 | 0.00028534 | 0.00042886 | 0.0005263 | 0.00027572 | 0.000674 | 0.00003988 | 0.00048543 | 0.00098551 | 0.00075018 |  |
|  | 0.00060165 | 0.00025159 | 0.00040761 | 0.00059476 | 0.00035393 | 0.00079916 | 0.00004262 | 0.00051994 | 0.00105947 | 0.00087933 |  |
|  | 0.00038565 | 0.0001728 | 0.00020841 | 0.0004044 | 0.00016579 | 0.00125116 | 0.00020055 | 0.00024618 | 0.00081306 | 0.00058261 |  |
|  | 0.00041237 | 0.00027468 | 0.00041891 | 0.00052309 | 0.00025339 | 0.00069844 | 0.00003303 | 0.00055882 | 0.00099095 | 0.00079381 |  |
|  | 0.00037096 | 0.00020801 | 0.00037457 | 0.00057217 | 0.00034163 | 0.00078104 | 0.00005602 | 0.00048008 | 0.00100357 | 0.00086871 |  |
|  | 0.00057354 | 0.00031649 | 0.00034688 | 0.0006527 | 0.00036367 | 0.00127832 | 0.00003124 | 0.00045981 | 0.00095576 | 0.00078986 |  |
| **Reference (R) (50 min)** | 0.0002535 | 0.00010953 | 0.00035401 | 0.00040379 | 0.00016659 | 0.00059267 | 0.00005416 | 0.00044906 | 0.00080209 | 0.00059498 |  |
|  | 0.00044825 | 0.0002377 | 0.00038674 | 0.00051964 | 0.00027638 | 0.00087848 | 0.00005432 | 0.0004813 | 0.00090593 | 0.00060813 |  |
|  | 0.00026343 | 0.00010714 | 0.00026165 | 0.00040751 | 0.00021333 | 0.00049226 | 0.00002483 | 0.00023701 | 0.00056704 | 0.00035892 |  |
|  | 0.00034831 | 0.00022334 | 0.0002978 | 0.00040845 | 0.00021857 | 0.00067075 | 0.00005575 | 0.00039322 | 0.00086018 | 0.00060283 |  |
|  | 0.00056272 | 0.00036723 | 0.00039093 | 0.00055644 | 0.0002812 | 0.00066534 | 0.00005824 | 0.00012576 | 0.00099152 | 0.00054923 |  |
|  | 0.00032286 | 0.00015728 | 0.00031782 | 0.00049286 | 0.00030017 | 0.00056546 | 0.00007623 | 0.0003135 | 0.00058901 | 0.00046565 |  |
|  | 0.0003071 | 0.00015346 | 0.0004947 | 0.00062167 | 0.00033178 | 0.00082958 | 0.00005058 | 0.00071908 | 0.00073401 | 0.00063135 |  |
|  | 0.00039117 | 0.00022048 | 0.0003198 | 0.00048226 | 0.00019103 | 0.0005964 | 0.00006926 | 0.00007575 | 0.00094992 | 0.00046297 |  |
|  | 0.00026241 | 0.00012163 | 0.00026914 | 0.00048388 | 0.00028062 | 0.00049347 | 0.00004962 | 0.00023828 | 0.00055028 | 0.00042744 |  |
| **"R" mean** | 3.51E-04 | 1.89E-04 | 3.44E-04 | 4.86E-04 | 2.51E-04 | 6.43E-04 | 5.4777E-05 | 3.37E-04 | 7.72E-04 | 5.22E-04 |  |
| **"S" mean** | 4.63E-04 | 2.41E-04 | 3.81E-04 | 5.57E-04 | 3.06E-04 | 9.08E-04 | 5.8073E-05 | 4.71E-04 | 9.64E-04 | 7.63E-04 |  |
| **"R" Standard deviation** | 1.02E-04 | 8.38E-05 | 7.30E-05 | 7.37E-05 | 5.54E-05 | 1.36E-04 | 1.4250E-05 | 1.99E-04 | 1.70E-04 | 9.63E-05 |  |
| **"S" Standard deviation** | 1.05E-04 | 5.18E-05 | 7.71E-05 | 7.66E-05 | 6.61E-05 | 2.77E-04 | 5.3920E-05 | 1.24E-04 | 7.00E-05 | 9.16E-05 |  |
| **Ratio S/R** | 1.32 | 1.28 | 1.11 | 1.15 | 1.22 | 1.41 | 1.06 | 1.40 | 1.25 | 1.46 |  |
|  |  |  |  |  |  |  |  |  |  |  |  |
| **Identified compounds** | Proline | Glutamate | Acetate | Citrulline | Arginine | Lysine | Valine | Leucine | Isoleucine |  | |
| **Samples / Buckets** | 2.085 | 2.055 | 1.895 | 1.875 | 1.725 | 1.435 | 1.035 | 0.955 | 0.935 |  | |
| **Stressed (S) (50 min)** | 0.00174583 | 0.00387141 | 0.00091529 | 0.000589 | 0.0006967 | 0.00120467 | 0.00333095 | 0.00174237 | 0.00178657 |  | |
|  | 0.00140053 | 0.00318965 | 0.00082848 | 0.00054315 | 0.00079499 | 0.00114166 | 0.00487306 | 0.00160358 | 0.00167791 |  | |
|  | 0.00135114 | 0.00260502 | 0.00067952 | 0.00054876 | 0.00087703 | 0.00100576 | 0.00706376 | 0.0016492 | 0.00178529 |  | |
|  | 0.00177856 | 0.00386048 | 0.00086648 | 0.000629 | 0.00079813 | 0.0013981 | 0.00309407 | 0.00198526 | 0.00217392 |  | |
|  | 0.00161776 | 0.00356124 | 0.00077709 | 0.00056864 | 0.00082544 | 0.00119259 | 0.00435861 | 0.00142852 | 0.00158606 |  | |
|  | 0.00101942 | 0.0022533 | 0.00078809 | 0.00043185 | 0.0006237 | 0.0007372 | 0.00672173 | 0.00197101 | 0.00205816 |  | |
|  | 0.00184075 | 0.00391844 | 0.00089156 | 0.00058963 | 0.00080244 | 0.00143819 | 0.0035438 | 0.00211094 | 0.00215176 |  | |
|  | 0.00163463 | 0.00358581 | 0.00076231 | 0.00055549 | 0.00064877 | 0.00119446 | 0.00468252 | 0.00156806 | 0.00168418 |  | |
|  | 0.00132183 | 0.00268183 | 0.00096713 | 0.00065248 | 0.00088094 | 0.00103864 | 0.00705814 | 0.00238154 | 0.0024716 |  | |
| **Reference (R) (50 min)** | 0.00253827 | 0.00597947 | 0.00104547 | 0.00071124 | 0.00069384 | 0.00135657 | 0.00115119 | 0.00194709 | 0.00160154 |  | |
|  | 0.002295 | 0.00544268 | 0.00103639 | 0.00075904 | 0.00076216 | 0.00117916 | 0.0012874 | 0.0015453 | 0.00132461 |  | |
|  | 0.00139067 | 0.00348871 | 0.00071271 | 0.00039467 | 0.00043421 | 0.0005651 | 0.00110841 | 0.0008322 | 0.00064793 |  | |
|  | 0.00239752 | 0.00535663 | 0.00127853 | 0.00094839 | 0.00087191 | 0.00128745 | 0.00105471 | 0.00204858 | 0.00155844 |  | |
|  | 0.00220247 | 0.00544139 | 0.00120463 | 0.00088603 | 0.00082137 | 0.00106007 | 0.001239 | 0.00160891 | 0.00134124 |  | |
|  | 0.00150236 | 0.00352819 | 0.00069961 | 0.00048601 | 0.00051421 | 0.0008634 | 0.00118815 | 0.00102782 | 0.00092886 |  | |
|  | 0.00236311 | 0.00551461 | 0.00091462 | 0.00061862 | 0.00067753 | 0.00114379 | 0.00099617 | 0.00165902 | 0.00120157 |  | |
|  | 0.00223292 | 0.00568521 | 0.00117882 | 0.00075942 | 0.00071942 | 0.00090485 | 0.00115812 | 0.00156208 | 0.0011603 |  | |
|  | 0.0013331 | 0.00317955 | 0.00081107 | 0.00049525 | 0.00051769 | 0.00088971 | 0.00120195 | 0.00111075 | 0.00105083 |  | |
| **"R" mean** | 2.03E-03 | 4.85E-03 | 9.87E-04 | 6.73E-04 | 6.68E-04 | 1.03E-03 | 1.15E-03 | 1.48E-03 | 1.20E-03 |  | |
| **"S" mean** | 1.52E-03 | 3.28E-03 | 8.31E-04 | 5.68E-04 | 7.72E-04 | 1.15E-03 | 5.02E-03 | 1.83E-03 | 1.93E-03 |  | |
| **"R" Standard deviation** | 4.77E-04 | 1.10E-03 | 2.15E-04 | 1.89E-04 | 1.49E-04 | 2.47E-04 | 9.04E-05 | 4.13E-04 | 3.02E-04 |  | |
| **"S" Standard deviation** | 2.68E-04 | 6.27E-04 | 8.87E-05 | 6.27E-05 | 9.40E-05 | 2.11E-04 | 1.60E-03 | 3.06E-04 | 2.96E-04 |  | |
| **Ratio S/R** | 0.75 | 0.68 | 0.84 | 0.84 | 1.16 | 1.12 | 4.35 | 1.23 | 1.61 |  | |

**Table S6**: XCMS parameters used for preprocessing of MS raw data in a Galaxy web-based environment^1^ .

| Processing | Parameters | Value |
| --- | --- | --- |
| xcmsSet | nSlaves | 6 |
|  | method | matchedFilter |
|  | step | 0.1 |
|  | fwhm | 30 |
|  | max | 5 |
|  | snthresh | 3 |
|  | steps | 2 |
| Group | method | density |
|  | sleep | 0.001 |
|  | minfrac | 0.5 |
|  | bw | 10 |
|  | mzwid | 0.1 |
|  | max | 5 |
| Rector | method | peakgroups |
|  | smooth | loess |
|  | extra | 1 |
|  | missing | 10 |
|  | span | 0.2 |
|  | family | gaussian |
| Group | method | density |
|  | sleep | 0.001 |
|  | minfrac | 0.5 |
|  | bw | 8 |
|  | mzwid | 0.25 |
|  | max | 5 |
| FillPeaks | method | chrom |

1. Giacomoni, F., Le Corguillé, G., Monsoor, M., Landi, M., Pericard, P., Pétéra, M., Duperier, C., Tremblay-Franco, M., Martin, JF., Jacob, D., Goulitquer, S., Thévenot, EA., Caron, C., Workflow4Metabolomics: a collaborative research infrastructure for computational metabolomics. *Bioinformatics*. **31**, 1493-1495, doi:10.1093/bioinformatics/btu813, (2015).


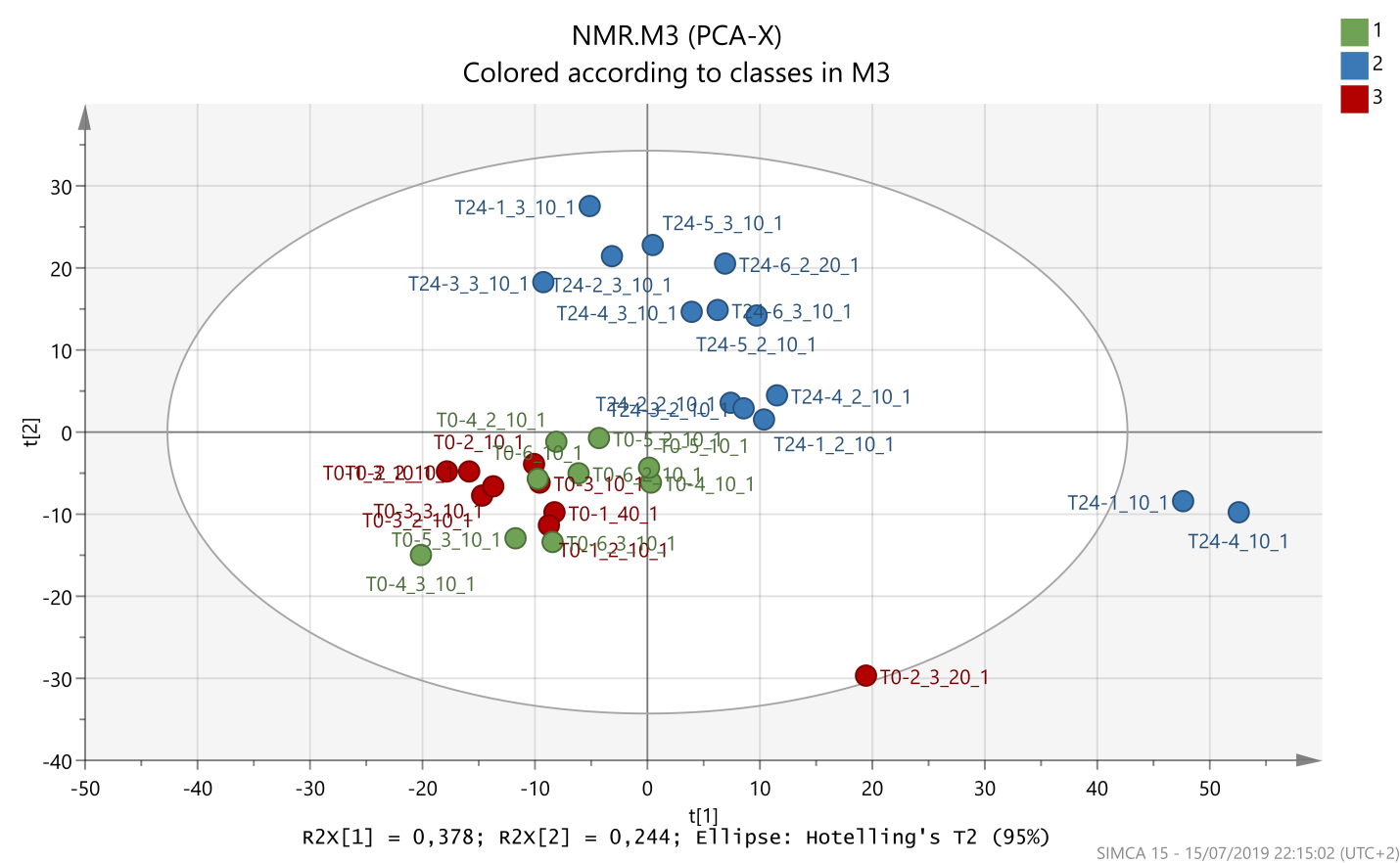

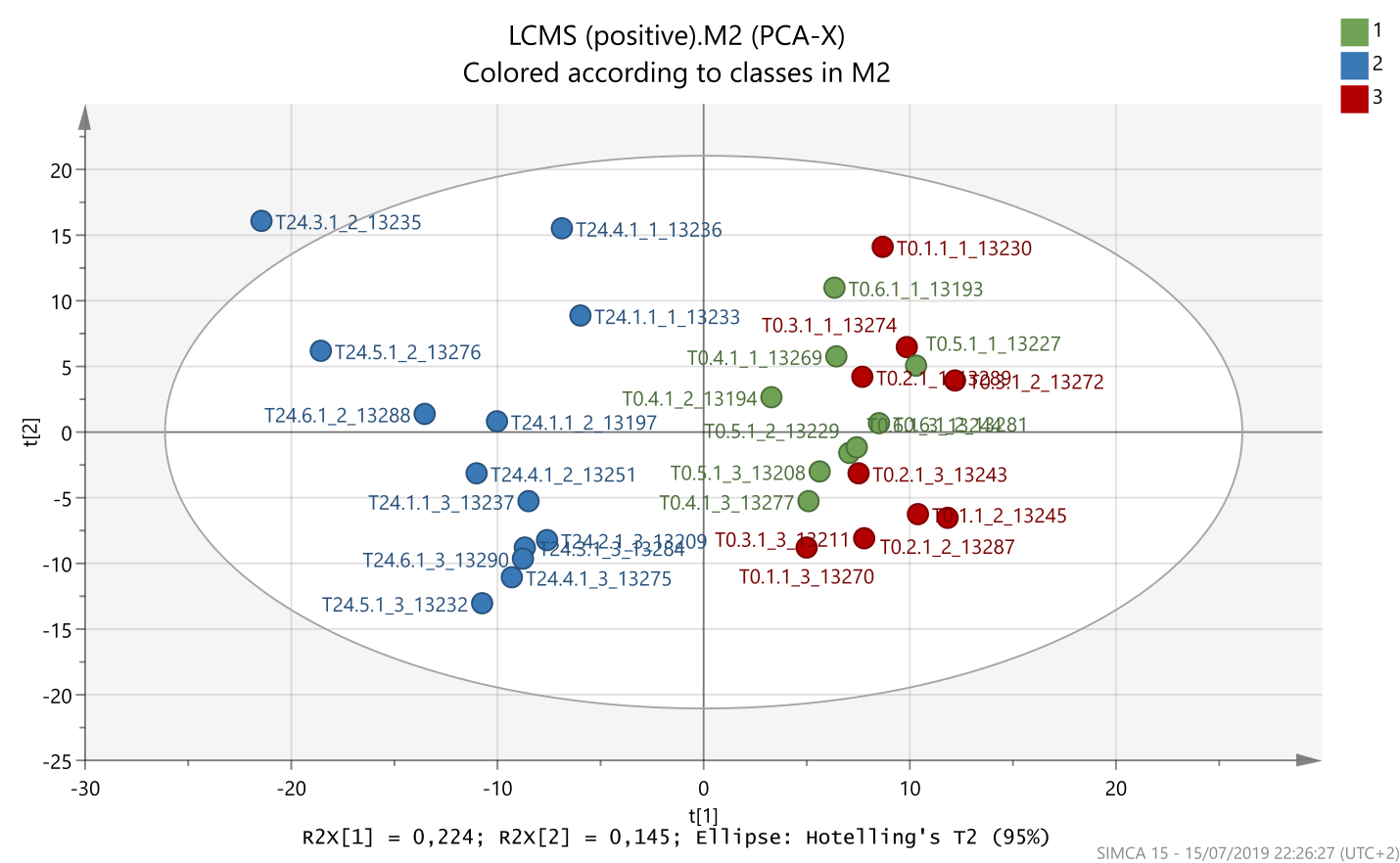


**Figure S1**: PCA analyses (UV scaling). Score plots obtained: (a) NMR and (b) LC-MS (example of the positive ionization mode. Red circles correspond to the samples incubated in the presence of H_2_O_2_ and extracted at 50 min; green circles correspond to the samples incubated in the absence of H_2_O_2_ and extracted at 50 min; blue circles correspond to the samples extracted at 24 hours with and without H_2_O_2_.


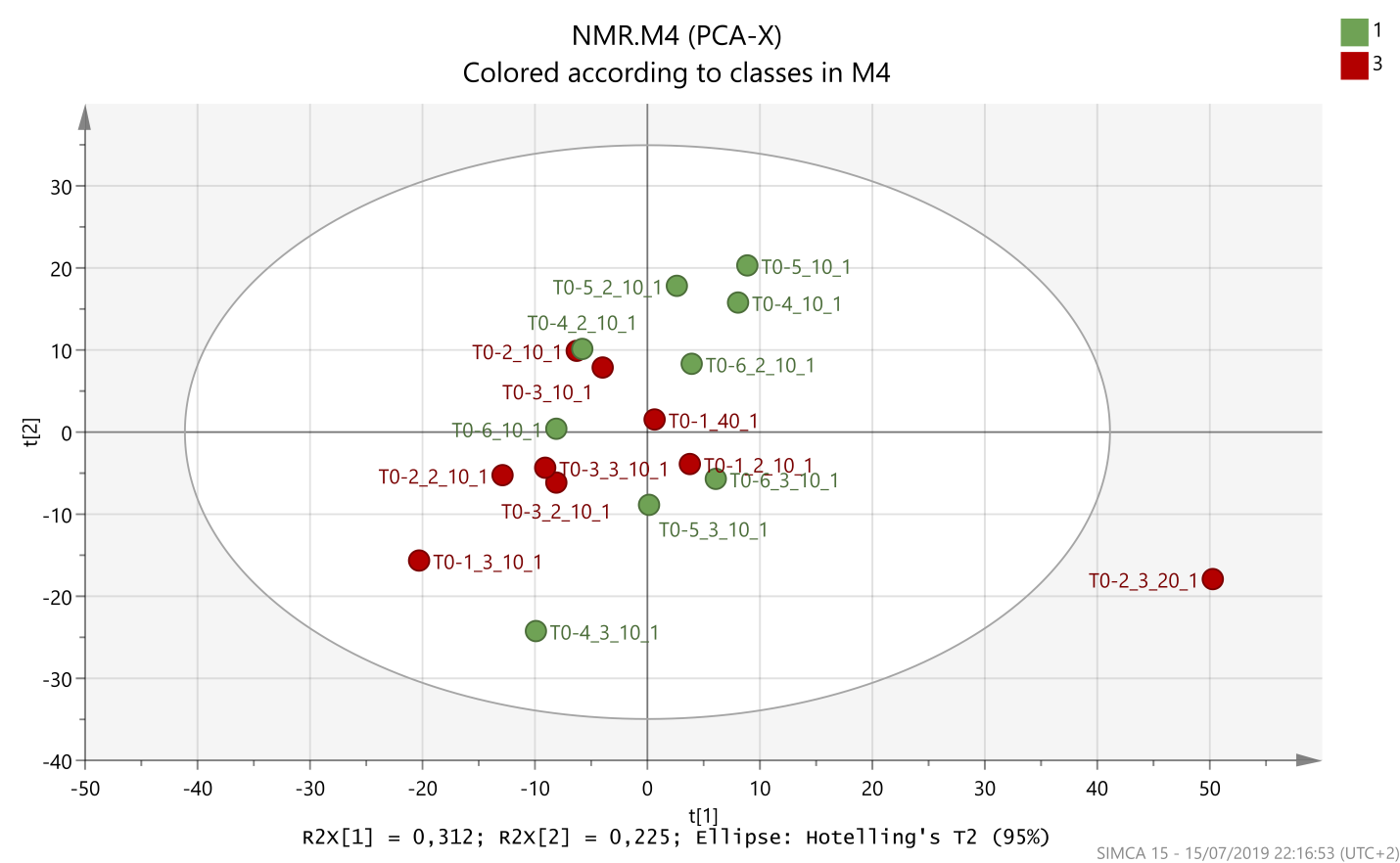

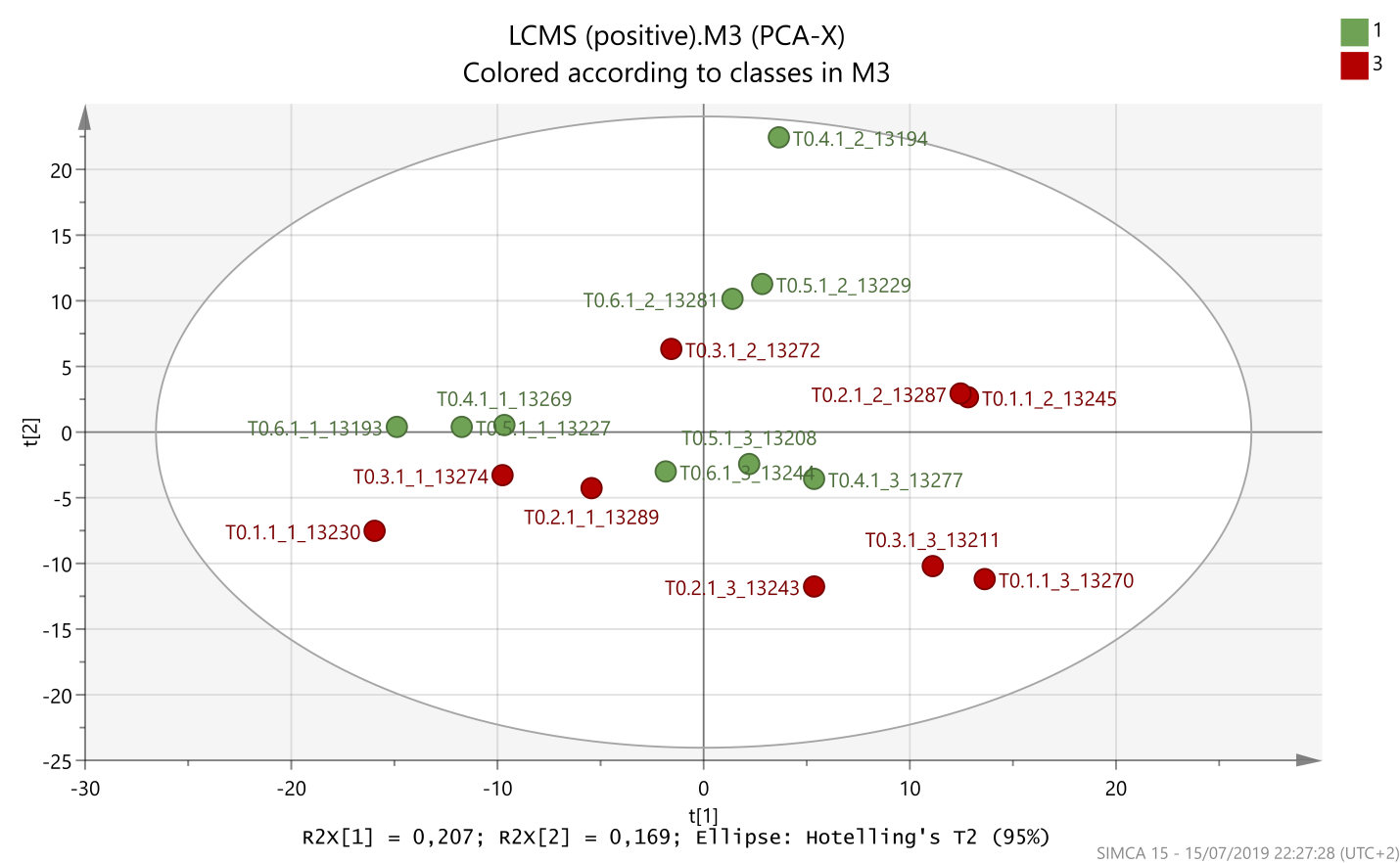


**Figure S2:** PCA analyses. Score plots (UV scaling) obtained for bacteria metabolomes extracted at 50 min of incubation in the presence (red circles) or in the absence (green circles) of H_2_O_2_: (a) NMR and (b) LC-MS (example of the positive ionization mode).


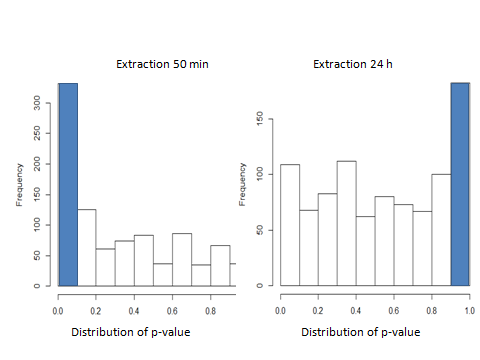


**Figure S3**: Distribution of data for the extraction at 50 min and 24 h in terms of p-value calculated using the statistical test of Wilcoxon-Mann-Whitney. This illustration represents the case of NMR analysis. Similar figure was obtained for LC-MS data for the two modes of ionization.

(a)


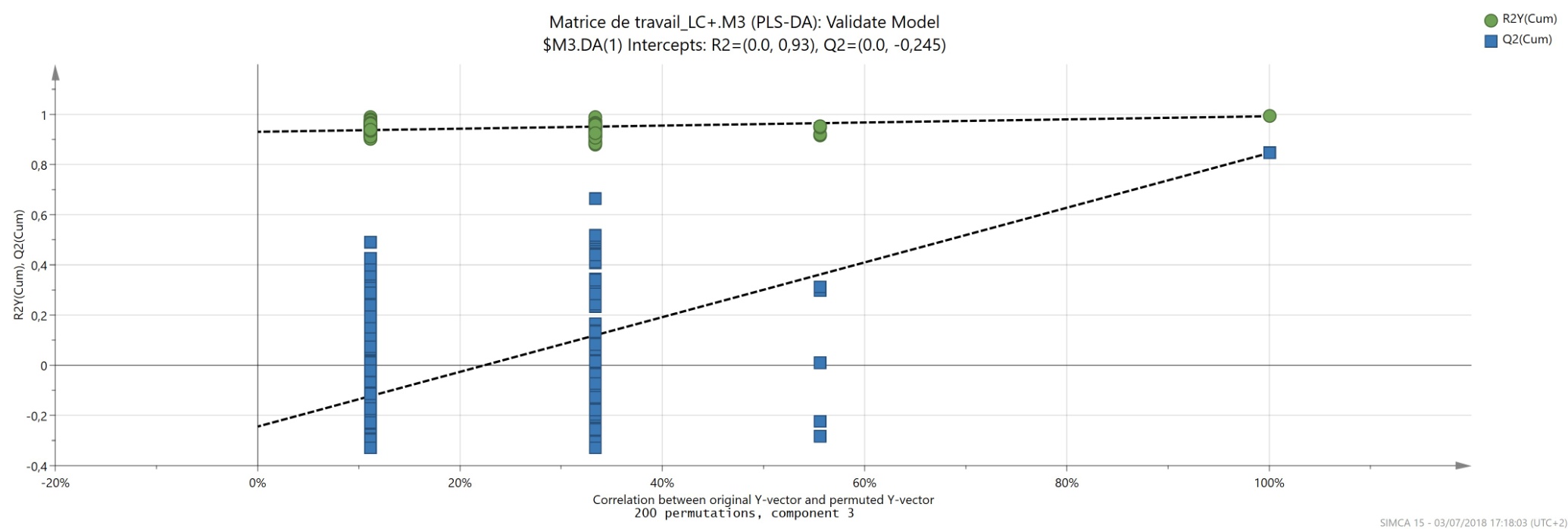


(b)


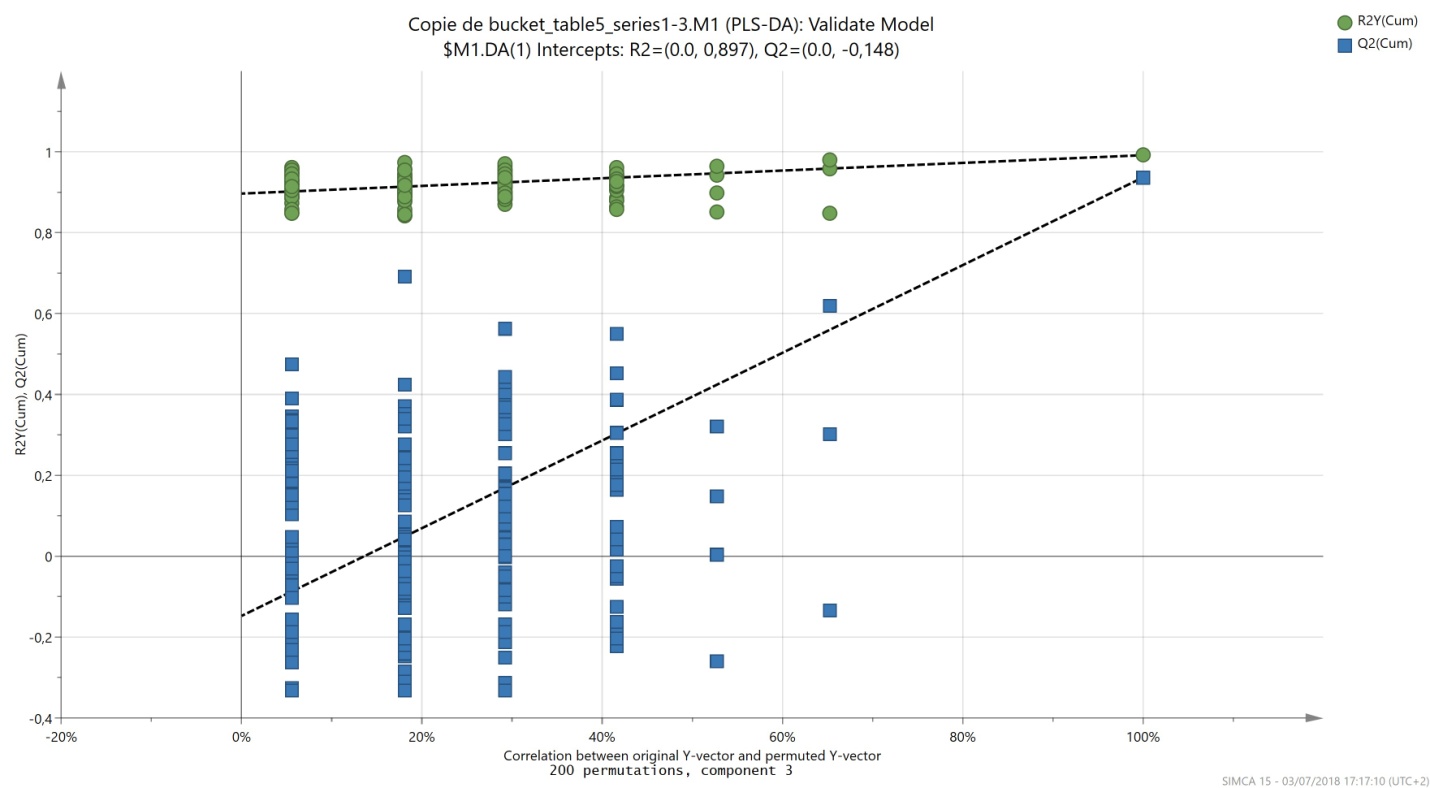


**Figure S4**: Visualization of the effect of 200 random permutations on model stability (R2 in green, Q2 in blue) (a) LC-MS positive mode (b) NMR.


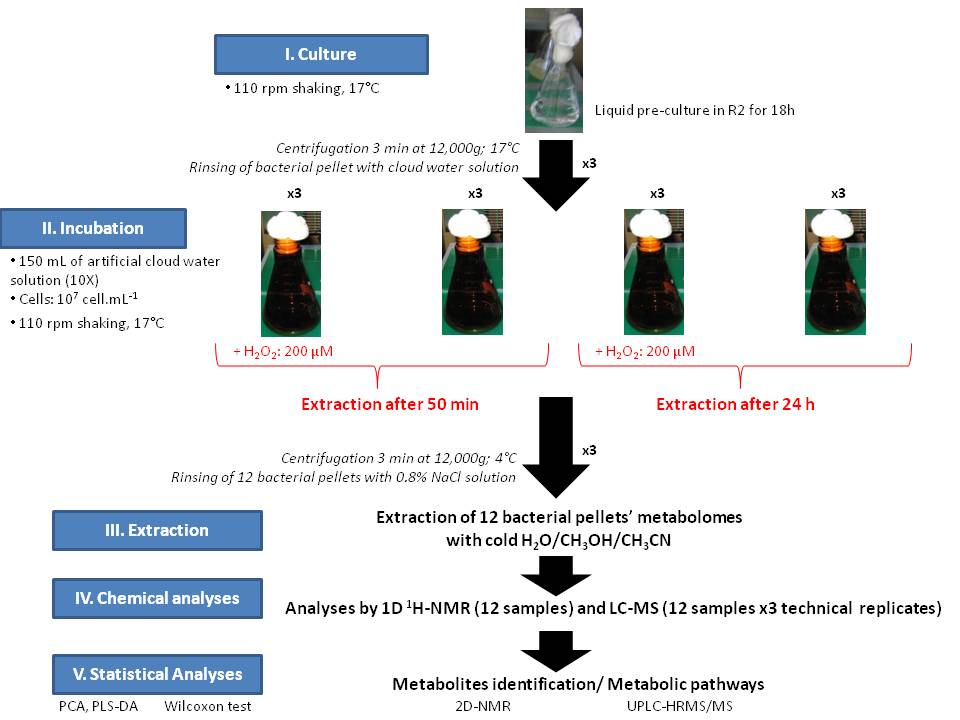


**Figure S5**: General workflow for the metabolomics experiments.

This protocol was repeated 3 times with 3 different liquid pre-cultures = **3 biological replicates= 12 erlenmeyers x 3.**

For LC-MS analyses technical samples were done. In total with the three biological replicates:

= **12 x 3 (biological replicates) x 3 (technical replicates)=technical samples.**
